# Advanced Design of Three-Dimensional Lithiophilic Carbon-Based Hosts for Anode-Free Lithium Metal Batteries

**DOI:** 10.1007/s40820-026-02229-1

**Published:** 2026-05-20

**Authors:** Fan Yang, Xiaoping Yang, Zhaoxia Xu, Maolin Zhang, Jiyue Hou, Shouyi Yuan, Yannan Zhang, Hao Wu, Yiyong Zhang, Fang Cheng

**Affiliations:** National and Local Joint Engineering Research Center for Lithium-Ion Batteries and Materials Preparation Technology, Key Laboratory of Advanced Battery Materials of Yunnan Province, School of Metallurgical and Energy Engineering, Kunming University of Science and Technology, Kunming, 650093 People’s Republic of China

**Keywords:** Anode-free lithium metal batteries, 3D lithiophilic carbon-based hosts, Heteroatom doping, Surface decorating, Lithiophilic gradient design

## Abstract

In recent years, anode-free lithium metal batteries (AFLMBs) have garnered significant attention as prospective next-generation high-energy batteries. However, the advancement of high-performance AFLMBs is severely impeded by challenges such as the uncontrolled growth of lithium dendrites and excessive volume expansion during cycling. Consequently, the design of three-dimensional (3D) lithiophilic carbon-based hosts to substitute conventional metal current collectors has emerged as a promising strategy for constructing high-performance AFLMBs. Herein, we present a critical and timely review of recent advances in the design of 3D lithiophilic carbon-based hosts for AFLMBs. Specifically, the intrinsic properties of various carbon materials, such as graphene, carbon nanotubes, porous carbon and carbon fibers, and their applications in AFLMBs, are first summarized. This is followed by a systematic classification of lithiophilic modification strategies, including surface heteroatom doping, surface decorating, and lithiophilic structural engineering and framework design, along with their research progress. Furthermore, the classifications, mechanisms, and structural configurations of lithiophilic gradient designs for 3D carbon-based hosts are summarized, with a specific focus on their application in high-performance AFLMBs. Finally, future challenges and perspectives regarding the lithiophilic structural design and practical application of novel 3D lithiophilic carbon-based hosts are proposed, offering valuable insights for the development of advanced AFLMBs.

## Highlights

- This review provides a systematic overview of advanced 3D lithiophilic carbon-based hosts, explicitly differentiating the design principles for anode-free lithium metal batteries (AFLMBs) from those for conventional LMB.
- Comprehensive lithiophilic modification strategies and their in-depth mechanisms are systematic classified and compared, with particular emphasis on lithium nucleation mechanisms and the impact of lithiophilic modification on lithium deposition behavior in AFLMBs.
- Future challenges and perspectives regarding the trade-offs between lithiophilic structural design and practical application of novel 3D lithiophilic carbon-based hosts are proposed, offering valuable insights for the development of next-generation.

## Graphical abstract

**Figure:**
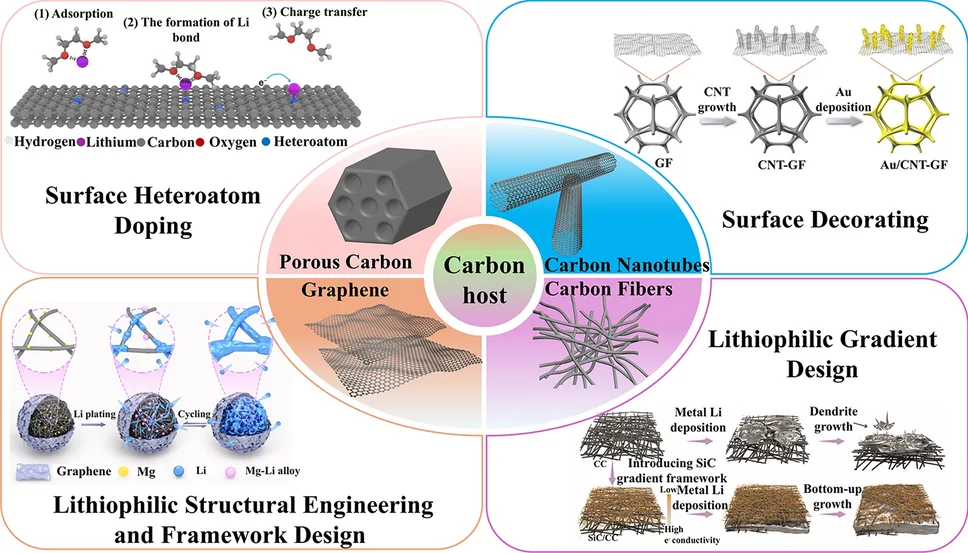

## Introduction

With the continuous advancement of energy-related technologies and the escalating demand for high-energy–density storage systems [1–3], lithium-ion batteries (LIBs) are increasingly unable to meet the growing application demands since the energy density of LIBs is approaching its intrinsic limit constrained by the low theoretical specific capacity of the graphite anode (372 mAh g⁻^1^) [4–6]. In this regard, lithium metal anodes (LMAs), possessing high theoretical specific capacity (3860 mAh g^−1^) and low redox potential (− 3.04 V vs. SHE), have emerged as a promising anode material for developing high-energy–density batteries [7–10]. This enables lithium metal batteries (LMBs) with metallic lithium as the anode to be foreground energy storage devices [11, 12]. Despite these salient advantages of LMAs, the following issues arising from charge–discharge cycles urgently require attention: 1) The uncontrolled growth of lithium dendrites may pierce the battery separators and poses potential safety hazards [13, 14], 2) the uneven lithium deposition and stripping on LMAs lead to nearly infinite volume expansion of the battery, which not only causes lithium dendrites to detach from the anode but also results in the accumulation of “dead lithium” [15–17], 3) the unstable solid electrolyte interphase (SEI) formed on LMAs continuously ruptures and reconstructs, consuming active Li and electrolyte, and ultimately causing rapid battery failure [18–20]. These issues jointly lead to low Coulombic efficiency (CE), short cycle life, and potential safety hazards in LMBs systems. Consequently, researchers have mostly focused their studies on the following aspects: 1) optimizing electrolyte composition or electrolyte additives [21–26], 2) constructing artificial SEI (ASEI) [27–32], 3) designing solid-state electrolytes (SSEs) [33–38]. Besides, our previous work also revealed that additive engineering and rational electrolyte design are favorable to build robust and durable SEI, thereby suppressing Li dendrites growth and achieving significant improvement in LMB performance [39–43]. However, due to the inherent safety hazards associated with lithium metal and the significant reduction in battery weight/volume energy density caused by the excessive use of lithium, the application of LMA is gradually replaced by anode-free design [3, 44–46].

Compared to LMBs, anode-free LMBs (AFLMBs) eliminate the direct use of LMAs and rely exclusively on the cathode lithium source. This type of battery is composed of a bare anode current collector (typically Cu foil) and a fully lithiated cathode. During charging, Li⁺ are extracted from the cathode and deposited on the Cu current collector. Upon subsequent discharging, the deposited lithium metal is stripped into the electrolyte and re-intercalated into the lattice of the cathode [47–53]. And thus, the advantages of AFLMBs include the following aspects: 1) A dramatic reduction in battery thickness and weight is achieved by omitting the anode material, thereby affording superior energy density (both gravimetrically and volumetrically) surpassing that of existing battery systems [54–57], 2) by replacing the LMA, potential safety hazards associated with highly reactive Li are avoided, and manufacturing costs are significantly reduced [45, 58, 59], 3) AFLMBs reflect the most realistic capacity loss mechanism in batteries and thus can be used to evaluate cycling performance [60]. In spite of these superiorities, AFLMBs suffer from uneven Li deposition and dendrite growth due to the lithiophobic nature of Cu current collectors [61–64]. Furthermore, planar Cu foils are lack of resilience against volume fluctuation during cycling, a factor that rapidly elevates internal pressure and surface roughness [65–68]. Surface coating and structural modification are effective approaches to address the poor lithiophilicity and volume elasticity of pristine Cu foils. On the one hand, surface coating enhances lithiophilicity by strengthening structural integrity, improving ionic conductivity and regulating Li⁺ flux [69–71]. On the other hand, Cu foils are structurally designed from planar structures into various 3D architectures with increased specific surface area (SSA) [72–75]. The key approaches encompass alloying [76–78], powder metallurgy [79–81], hydrogen bubble template [82–84], 3D printing [85–88], femtosecond laser [89–91], magnetron sputtering [92–94], photolithography [95–97], sulfide reduction [98–100], and phase transformation [101–103], etc. Although the above strategies improve the performances of AFLMBs, most fabrication processes for modified Cu collectors are time-consuming, costly, and complex. Consequently, significant challenges remain regarding their scalability and cost-effectiveness before their practical application in the battery industry. In contrast with metal-based collectors, carbon-based materials better offset these drawbacks, offering excellent conductivity, low cost, high stability, and strong mechanical strength [104–107].

In recent years, various carbon-based materials have been designed for AFLMBs, such as grapheme [108–110], carbon nanotubes (CNTs) [111–113], porous carbon (PC) [114, 115], carbon fibers (CFs) [116, 117], and carbon cloth (CC) [118, 119], etc. Given the host-free nature of LMAs and the significant volume variation it undergoes during repeated plating and stripping, carbon-based materials with abundant three-dimensional (3D) porous structures are conducive to accommodating Li deposition on the surface and within the pores of carbon materials [120–123]. Additionally, the electronic conductive networks of carbon-based materials facilitate electron–electrode contact. Consequently, current collectors optimized with carbon-based materials can be expected to deliver superior performance during battery evaluation. Nevertheless, apart from reduced graphene oxide (rGO), which exhibits a certain degree of lithiophilicity, other carbon-based materials do not display intrinsic lithiophilicity [119, 124]. Hence, there is a need to perform lithiophilic modification on carbon-based materials to strengthen their lithium affinity and attain enhanced Li deposition behavior. For instance: 1) Surface heteroatom doping [125], which can be realized by doping highly lithiophilic heteroatoms onto the surface of carbon-based materials (i.e., N [120, 126, 127], P [128], etc.), constructs lithiophilic active sites and guides uniform Li deposition along the 2D plane direction of the carbon hosts, 2) decorating the surface of carbon-based materials with other highly lithiophilic metals or metal oxides, such as Au [129], Ag [130], Zn [131], ZnO [132], MgO [133], etc., promotes the uniform nucleation and deposition of lithium, 3) 3D lithiophilic porous framework design, which affords structural support, spatial buffering, and abundant Li deposition sites, enables the suppression of lithium dendrites and volume fluctuation [134–137], 4) constructing a lithiophilic gradient inside carbon-based substrates. Taking carbon-based materials as the framework, this proposal distributes lithiophilic sites in a gradient manner inside to direct the bottom-up deposition of metallic lithium within the framework, thereby suppressing lithium dendrite growth and volume expansion [2, 138–140].

**Fig. 1:**
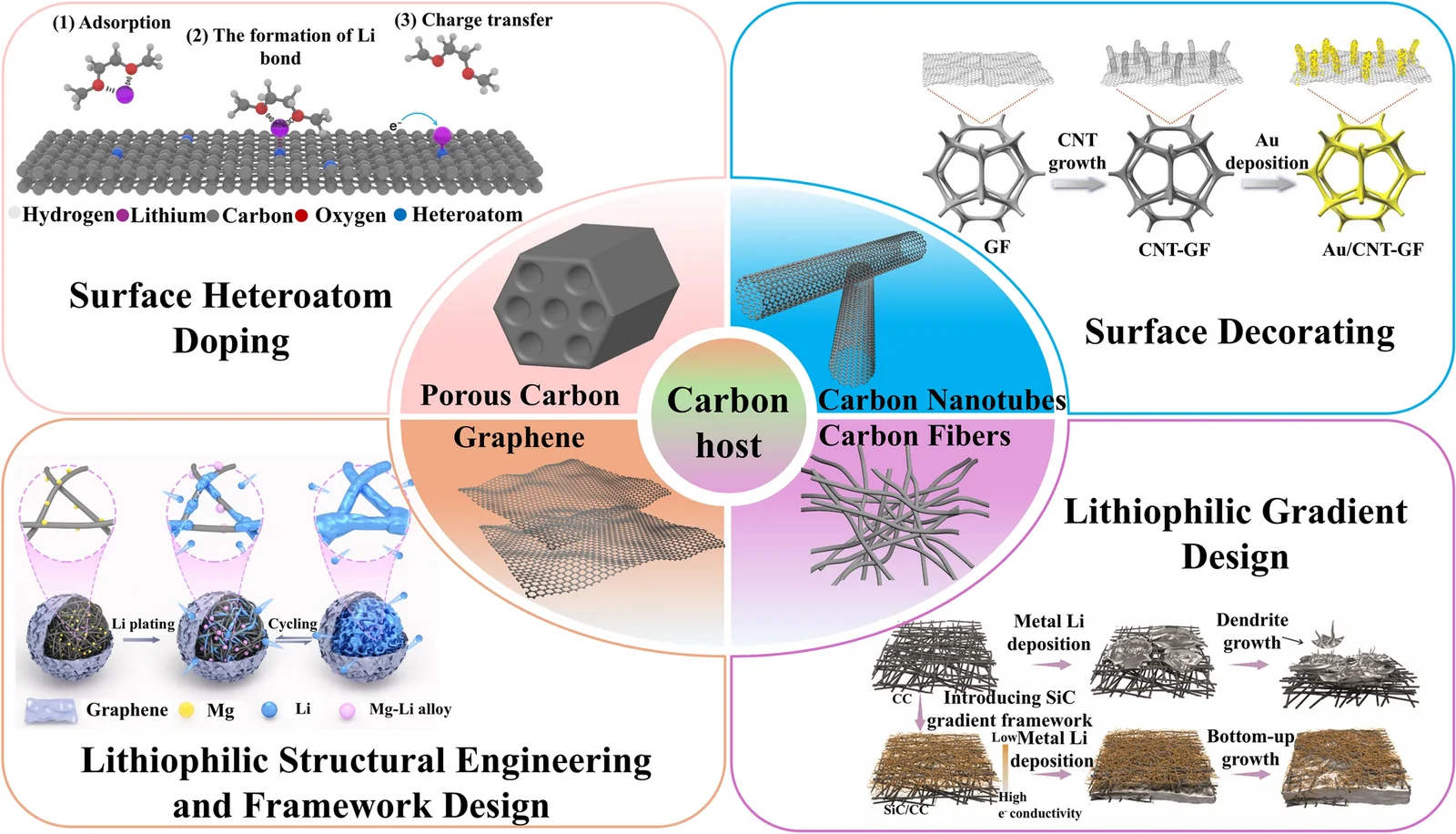
Schematic outline of major content discussed in the present review. This review systematically summarizes the structural and performance characteristics of various carbon-based hosts and the main lithiophilic modification strategies: surface heteroatom doping [125], Copyright 2019 The American Association for the Advancement of Science; surface decorating [129], Copyright 2022, Elsevier; lithiophilic structural engineering and framework design [120], Copyright 2024, Elsevier; lithiophilic gradient design [141], Copyright 2022, Elsevier

Although the strategies mentioned above are critical for high-performance AFLMBs, most existing reviews treat them as a subset of general LMBs or 3D carbon materials, thereby overlooking the distinct challenges posed by the anode-free configuration. Specifically, unlike conventional LMBs where excess lithium acts as a buffer, AFLMBs operate with a finite lithium inventory and lack an initial nucleation template, rendering the lithiophilicity and interfacial stability of the host far more critical than in traditional systems. Consequently, a review focusing exclusively on the specific requirements of AFLMBs is urgently needed to bridge this gap. In this context, this work provides a systematic overview of advanced 3D lithiophilic carbon-based hosts, explicitly differentiating the design principles for AFLMBs from those for conventional LMBs. We first summarize the structural characteristics of carbon-based materials and categorize lithiophilic modification strategies, including heteroatom doping, surface decorating, and structural design (Fig. 1), specifically based on their efficacy in regulating initial nucleation and minimizing irreversible lithium loss. Furthermore, we critically discuss the mechanisms and structural configurations of gradient lithiophilic designs, highlighting their role in managing local current density within the limited anode space. Finally, we address key challenges in transitioning from structural design to practical applications, such as scalable fabrication and cost control, and propose future directions to guide the rational design of next-generation 3D carbon hosts for AFLMBs.

## Nucleation Mechanism and Lithiophilic Site Modulation

During the cycling of AFLMBs, lithium metal is repeatedly deposited onto and stripped from the current collector. This process leads to two major challenges: lithium dendrite growth and infinite volume changes. Accordingly, this section discusses the prevailing lithium nucleation mechanisms and the impact of lithiophilic modification on lithium deposition in AFLMBs.

### Nucleation Thermodynamics and Li⁺ Transport Kinetics

**Fig. 2:**
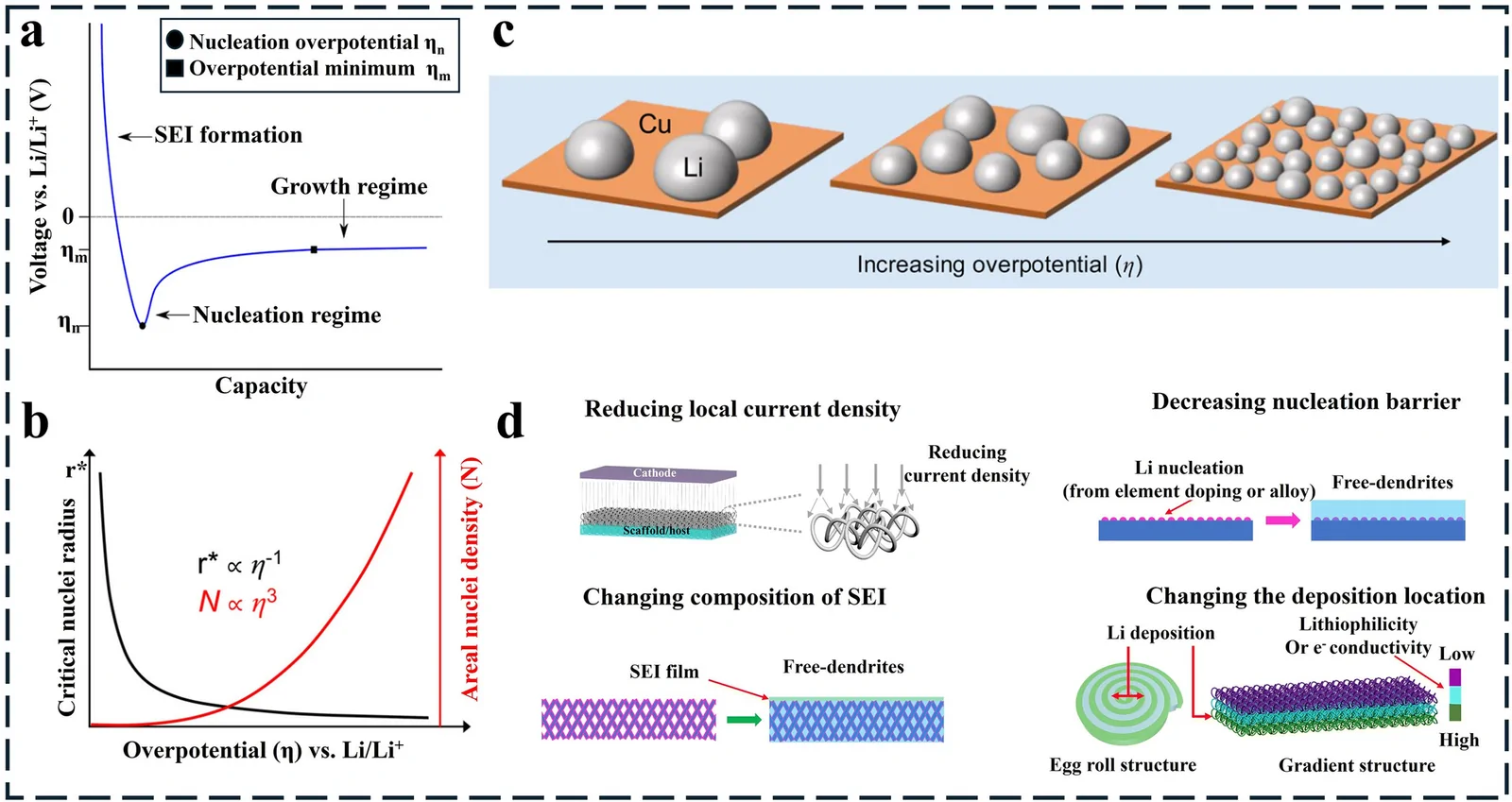
**a** Schematic plot showing a typical voltage profile of galvanostatic Li deposition [143]. Copyright 2019, American Chemical Society. **b** Schematic plot of the dependence of critical Li nuclei radius and areal nuclei density on the overpotential of Li deposition. **c** Schematic illustrating the size and density of Li nuclei deposited on Cu at varying overpotentials [142]. Copyright 2017 American Chemical Society. **d** Schematic diagram of strategy for mitigating lithium dendrite growth by extending Sand's time [150]. Copyright 2024 Royal Society of Chemistry

Based on classical nucleation theory, the nucleation process of lithium involves a corresponding free energy barrier, which is associated with the thermodynamic penalty required to form critical atomic clusters. In electrodeposition, modulating the overpotential of the reduction reaction can alter the electrochemical supersaturation at the working electrode, thereby effectively regulating this nucleation barrier [142]. The magnitude of the voltage spike at the initial stage of lithium deposition is defined as the nucleation overpotential (*η*_*n*_), and the additional voltage required to sustain the deposition and growth of lithium structures after the initial nuclei formed is defined as the growth overpotential (*η*_*p*_). The difference between these two values (*η*_*p*_-*η*_*n*_) characterizes the nucleation overpotential of the substrate, and its magnitude directly indicates the difficulty level of lithium nucleation on that substrate [142, 143] (Fig. 2a). Under ideal conditions, uniform lithium nucleation can be achieved. According to the homogeneous nucleation equation [144, 145], the applied current density during electrodeposition can be correlated with the critical nucleus size of lithium. According to classical nucleation theory, the Gibbs free energy change (ΔG_nucleation_) for forming a spherical nucleus of radius r is the sum of the volume free energy and the surface free energy, as expressed in Eq. (1) [146].

1 $$\Delta {G}_{nucleation}=-\frac{4}{3}\pi {r}^{3}\Delta {G}_{v}+4\pi {r}^{2}\gamma $$

where ΔG_v_ (J m⁻^3^) is the free energy change per unit volume and γ (J m⁻^2^) is the surface energy of the lithium–electrolyte interface. The relationship between ΔG_v_ and the deposition overpotential (*η*) is given by Eq. (2) [147].

2 $$\Delta {G}_{v}=F\left|\eta \right|/{V}_{m}$$

where F is the Faraday constant (96,500 C mol⁻^1^) and Vₘ is the molar volume of Li (m^3^ mol⁻^1^). Therefore, the critical radius (r_crit_) for lithium nucleation can be derived, as shown in Eq. (3) [147]. According to Eq. (3), nuclei with a radius larger than r_crit_ will survive and continue to grow, while smaller ones will dissolve into the electrolyte [2].

3 $${r}_{crit}=2\gamma {V}_{m}/F\left|{\eta}_{n}\right|$$

From Eq. (3), it is evident that r_crit_ is inversely proportional to *η*_*n*_. Consequently, the lithium nuclei density (N) is proportional to η_n_^3^ (Fig. 2b). Furthermore, according to Butler–Volmer electrode kinetics, the current density is proportional to the overpotential (see Eq. (4)) [148]. As a consequent, at low current densities, lithium nucleation within the region tends to be larger and sparser, whereas at high current densities, the nucleation becomes smaller and more densely distributed (Fig. 2c).

4 $$ j = j_{0} \left[ {{\mathrm{exp}}\left( {\frac{{\alpha _{a} F\eta }}{{RT}}} \right) - {\mathrm{exp}}\left( { - \frac{{\alpha _{c} F\eta }}{{RT}}} \right)} \right] $$

where j is the current density, j₀ is the exchange current density, αₐ and α_c_ are the anodic and cathodic transfer coefficients, respectively, η is the overpotential, and F, R, and T represent the Faraday constant, gas constant, and temperature, respectively.

The aforementioned thermodynamic principles of nucleation outline the nucleation and growth mechanism of lithium. However, to fully understand the formation of lithium dendrites, it is essential to examine the kinetics of Li⁺ transport. To date, the Sand model proposed by Professor J. N. Chazalviel remains the most widely accepted model for describing lithium dendrite growth [149]. This model indicates that dendrites form when the Li⁺ concentration at the anode surface drops to zero due to the increase in current density, a phenomenon known as Sand's behavior. The time required for the Li⁺ concentration to reach zero is defined as the Sand's time (τ), as expressed in Eq. (5).

5 $$\tau =\pi D[{e}^{2}{C}_{0}^{2}{\left({\mu}_{a}+{\mu}_{{Li}^{+}}\right)}^{2}/{\left(2J{\mu}_{a}\right)}^{2}]$$

where μ_a_ and μ_Li_^+^ are the mobilities of anions and Li⁺ ions, respectively. C₀ is the initial Li⁺ concentration, and J is the current density. D represents the ambipolar diffusion coefficient, which is related to the diffusion coefficients and mobilities of Li⁺ cations and anions.

Based on the aforementioned Sand model, lithium dendrites form under high current density or low ionic diffusion coefficients. Therefore, strategies such as constructing a multidimensional host to reduce localized current density, applying surface modifications to lower the nucleation overpotential of Li, stabilizing the SEI structure to accelerate Li⁺ transport, and designing host architectures to alter Li⁺ deposition sites can effectively prolong Sand's time [150] (Fig. 2d), thereby mitigating dendrite growth in AFLMBs.

### Lithium Failure Mechanisms

Lithium inventory loss serves as a core quantitative metric for performance degradation in LMBs and especially AFLMBs. It primarily refers to the irreversible loss of active lithium during electrochemical cycling, rendering it unavailable for subsequent electrochemical reactions. This ultimately manifests as capacity decay, reduced CE and shortened cycle life. The primary loss mechanisms stem from SEI instability, lithium dendrite growth and dead lithium formation. Therefore, the primary lithium failure mechanisms of AFLMBs are elaborated in this section.

#### SEI Dynamic Evolution

**Fig. 3:**
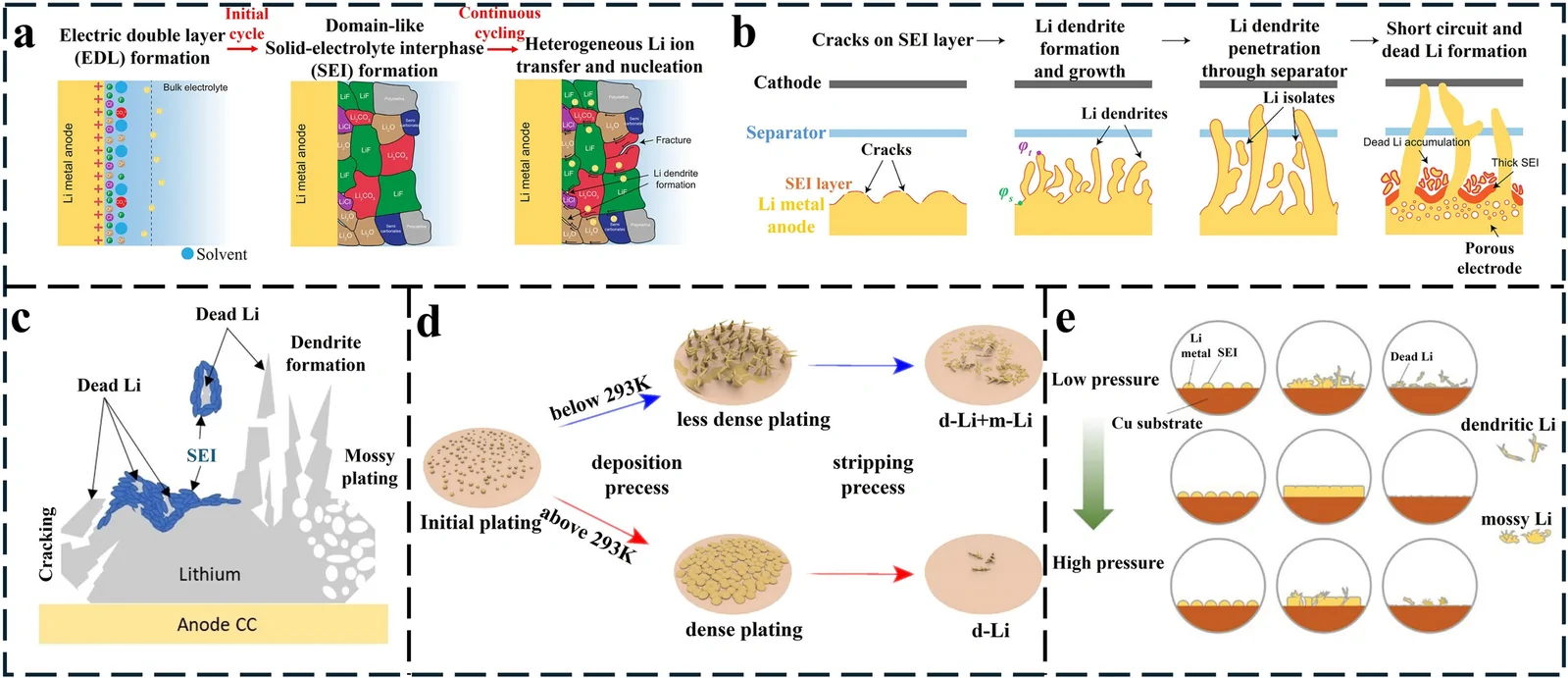
**a** Schematic illustration of SEI layer. **b** Schematic illustrations of Li dendrite growth and dead Li formation [158]. Copyright 2023 Wiley. **c** Mechanisms of dead lithium formation [170]. Copyright 2024 Wiley. **d** Schematic illustrations of the effect of temperature on lithium metal deposition behavior and dead lithium formation [171]. Copyright 2025 Wiley. **e** Schematic representation of the effect of stacking pressure on lithium metal deposition and dead lithium formation [175]. Copyright 2024 Wiley

Lithium metal exhibits exceptionally high chemical reactivity owing to its ultra-low electrochemical potential. Upon contact with liquid electrolyte, it rapidly reacts, leading to the in situ formation of the SEI layer on the LMA surface. This unique SEI layer passivates the lithium metal surface, thereby suppressing continuous parasitic reactions between LMA and electrolytes [151, 152]. According to the early proposed dual-layer structure of the SEI, it consists of a thin but dense layer adjacent to the electrode and a thick but porous layer on the electrolyte side [153]. Subsequently, building upon this theory, researchers proposed a mosaic structure for the SEI, wherein the SEI consists of multiple inorganic and organic layers. The thin, dense layer adjacent to the electrode contains stable anions derived from the electrolyte salt, such as O^2^⁻, F⁻, and Cl⁻. During Li deposition, these anions combine with Li⁺ to form inorganic lithium compounds, including Li₂O, Li₂Cl, and LiF [154, 155], filling the SEI layer on the electrode surface. In addition, organic solvents such as ethylene carbonate (EC) and diethyl carbonate (DEC) undergo reduction in regions farther from the electrode, resulting in the formation of insoluble SEI components, including Li₂CO₃, semi-carbonates, and oligomeric organic compounds such as polyolefins (Fig. 3a) [156–158]. Therefore, these surface heterogeneities accelerate the formation of lithium dendrites and the passivation layer on the LMA [159]. It is worth noting that the SEI is not a static layer; it undergoes continuous dynamic evolution during battery cycling. This is due to the significant volume changes of the LMA upon cycling, which renders the SEI susceptible to fracture under mechanical stress. Once fractured, fresh lithium is exposed and reacts again with the electrolyte, generating a new SEI. This cyclic fracture-repair process leads to SEI instability [160, 161]. Furthermore, the different components of the SEI exhibit varying Li⁺ transport kinetics and are heterogeneously distributed, leading to a non-uniform Li⁺ flux across the SEI. This uneven Li⁺ distribution readily induces non-uniform lithium deposition and promotes dendrite formation [150]. Extended cycling leads to the complete consumption of the electrolyte and a decline in CE [162], ultimately leading to battery failure.

The transition from LMBs to AFLMBs represents a shift from a forgiving system to a strict system. In LMBs, a host design that yields 99% CE is often acceptable due to the lithium reservoir. In AFLMBs, the same 99% CE limits cycle life to fewer than 100 cycles. Therefore, AFLMB host design is not an incremental improvement but a pursuit of near-perfect reversibility (> 99.9% CE). This necessitates fundamentally different selection criteria for carbon hosts, prioritizing interfacial compatibility and minimal dead lithium over simple structural stability. A fundamental distinction lies in the first plating cycle. In LMBs, lithium is stripped from a foil and replated onto the host. The surface chemistry is then established. In AFLMBs, the host must induce the initial nucleation of lithium ions from the cathode onto a bare current collector. This requires a much higher degree of lithiophilicity to overcome the high nucleation barrier of carbon materials, a factor less critical in LMBs where the host is often pre-lithiated or used with liquid lithium.

#### Lithium Dendrite Growth

During the unlimited charge–discharge cycles, lithium metal undergoes repeated deposition and stripping. Particularly during charging, lithium nuclei grow on the anode surface and develop into dendrites, which typically exhibit needle-like, mossy, whisker-like, or dendritic morphologies [158]. Monroe et al. [163] indicate that a potential difference exists between the base potential (*φₛ*) and the tip potential (*φₜ*) of dendrites, with the radius of curvature of the dendrite being inversely proportional to the tip potential. Consequently, lithium nuclei with smaller radii of curvature exhibit higher tip potentials. Moreover, the dendrite tip surface possesses a high density of steps and kinks, which endows this region with a higher exchange current density, thereby reducing the energy activation for lithium deposition. As a result, the local overpotential at the dendrite tip is lower than that on flat regions, further promoting preferential lithium deposition at the tip [164]. Moreover, the potential difference (*φ*_*t*_-*φ*_*s*_) constitutes the driving force for dendrite growth. Additionally, the lower deposition overpotential at the tip makes Li preferentially deposit along protrusions [165, 166], as dendrites extend, they may eventually pierce the separator and contact the cathode, leading to short circuits and even thermal runaway [158] (Fig. 3b).

#### Dead Lithium Formation

In AFLMBs, one of the most critical challenges hindering the practical application of AFLMBs is the formation of “dead Li” that becomes electrically isolated from the current collector due to uneven deposition and stripping. In specific, the repeated deposition/stripping of lithium at the anode induces volume expansion, leading to the formation of irregular cracks in the SEI [167]. These cracks expose fresh Li metal to the electrolyte, creating hotspots for subsequent Li dendrite growth through the fractured regions. In subsequent cycles, this process further promotes Li dendrite formation. Ultimately, due to the irregular surface structure of the SEI, dissolution occurs at the irregular and elongated roots, causing a portion of the Li dendrites to become electrically isolated from the anode, thereby forming dead lithium [158, 168–170] (Fig. 3c). The accumulation of dead Li leads to rapid capacity decay and increased cell impedance [171, 172]. Recent studies have focused on understanding the dynamic evolution of dead Li and developing strategies to reactivate or minimize it [170]. In the context of carbon-based hosts, structural engineering plays a vital role in regulating Li nucleation to ensure uniform deposition, thereby fundamentally reducing the generation of dead Li. Furthermore, a lithiophilic framework can maintain good electrical contact with the deposited Li, preventing its isolation during cycling.

Meanwhile, the formation of dead lithium is influenced by various factors, with temperature being one of the critical factors. In their study of ether-based electrolyte AFLMBs, Liu et al. [171] found that when the temperature drops below 293 K, the SEI becomes enriched with lithium oligoethylene oxide (LOCO), which exhibits low ionic conductivity. This leads to uneven current distribution, induces lithium dendrite growth and results in substantial dead lithium formation. In contrast, at temperatures above 293 K, the LOCO content decreases, leading to an improved ionic conductivity of the SEI. Lithium deposition becomes more compact, and dead lithium is significantly reduced (Fig. 3d). In addition to temperature, the properties of the SEI also play a crucial role in dead lithium formation. Yoon et al. [172] demonstrated that a preformed SEI can effectively inhibit lithium dendrite growth. However, the extent of this effect depends on its chemical composition. Inorganic SEI layers rich in LiF and Li_3_N effectively suppress dendrite growth and reduce dead lithium formation. In contrast, thick SEI layers rich in organic components (such as Li_2_CO_3_ and Li_2_O) exacerbate dead lithium formation due to their high resistance and inhomogeneity. To gain a deeper understanding of the growth mechanism of dead lithium, a range of quantitative methods such as titration gas chromatography (TGC) [173] and in situ nuclear magnetic resonance (NMR) [168] have proved highly effective in observing dead lithium formation [170]. Grey's group revealed that in situ NMR enables real-time, non-destructive quantitative analysis of dead lithium formation and evolution [174]. Lin et al. [175] investigated the effect of stack pressure on dendrite and dead lithium growth using in situ NMR, revealing that lithium dendrites grow rapidly under low pressure, whereas mossy lithium forms under high pressure (Fig. 3e). Fang et al. [176] utilized titration gas chromatography (TGC) to measure the amount of gas generated from the reaction between dead lithium and specific protic solvents, enabling precise quantification of residual Li⁰ content in the electrode and thereby distinguishing dead lithium from Li⁺ loss within the SEI. Additionally, to understand the formation mechanisms of dead lithium in different electrolytes, they employed cryo-electron microscopy to examine the microstructure and nanostructure of unreacted metallic lithium. Furthermore, electrochemical methods such as the Aurbach method can indirectly assess the irreversible capacity loss caused by dead lithium through analysis of lithium stripping/deposition efficiency [172].

### Impact of Lithiophilic Modification on Lithium Deposition, SEI Stability, and Dead Lithium Formation

**Fig. 4:**
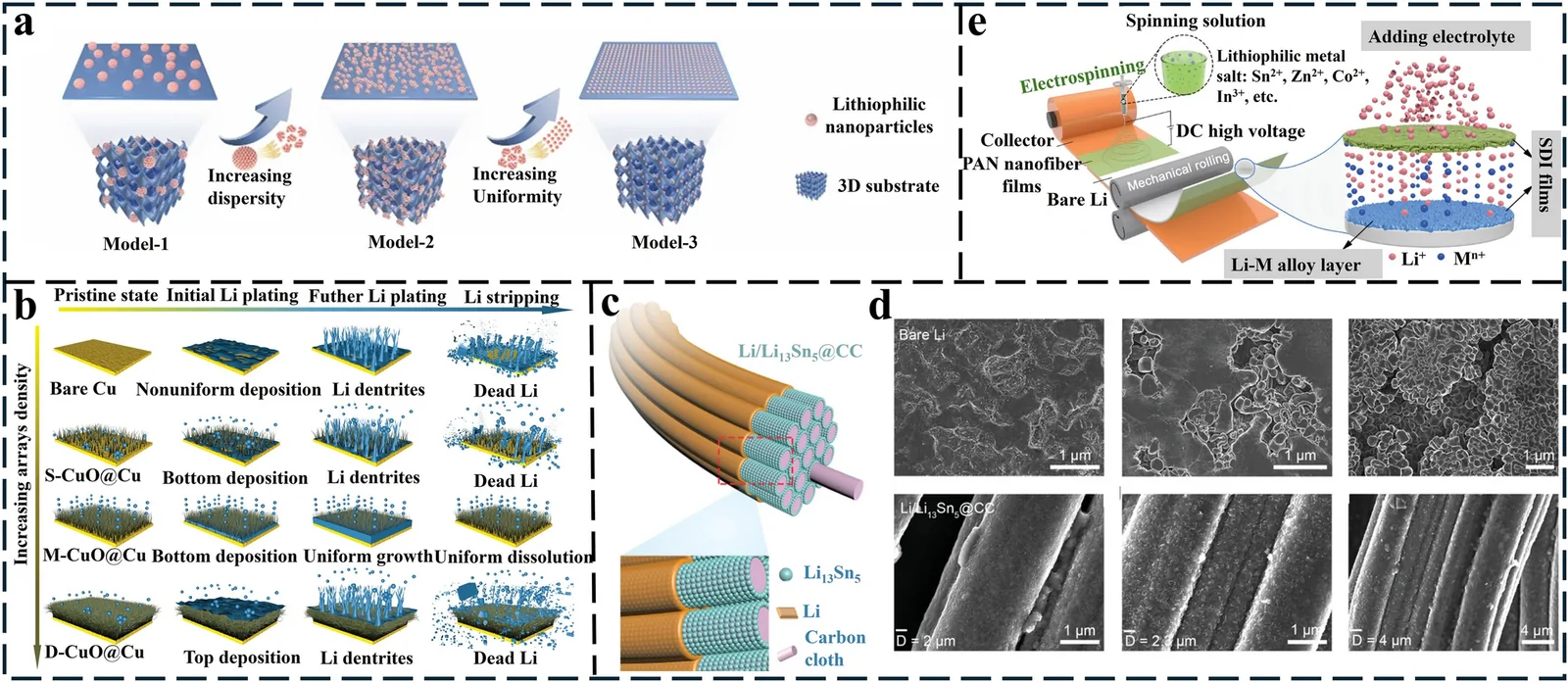
**a** Illustrations of the substrates quantitatively modeled by different dispersity of lithiophilic nanoparticles [177]. Copyright 2021 Elsevier. **b** Schematic illustration of the Li deposition patterns on bare Cu, S-CuO@Cu, M-CuO@Cu, and D-CuO@Cu electrodes [178]. Copyright 2023 Wiley. **c** Schematic illustration of Li_13_Sn_5_@CC and the associated Li deposition. **d** SEM images of Li stripping on bare Li (top) and Li/Li₁₃Sn₅@CC (bottom) with Li deposition capacities of 0, 5, and 10 mAh cm⁻^2^ from left to right [179]. Copyright 2024 Wiley. **e** Schematic illustration of the preparation of SDI-coated Li anode [180]. Copyright 2023 Wiley

During lithium deposition and stripping, maintaining a uniform Li⁺ flux across the entire electrode surface is critically important. To address this, various research groups have implemented lithiophilic modifications on host surfaces, which promote uniform lithium deposition across multiple scales influencing lithium deposition kinetics, SEI stability, and dead lithium formation. Firstly, initial Li nucleation and deposition usually present a lithiophilic site-dependent behavior. Using a nonlinear phase-field approach based on Butler–Volmer reaction kinetics, Liu et al. [177] simulated lithium deposition on model substrates and revealed that sparsely distributed nanoparticles tend to form isolated Li nuclei during initial cycling. These isolated nuclei induce vertical Li growth toward the electrolyte, eventually leading to severe concentration polarization at the electrode–electrolyte interface and the formation of Li dendrites. Subsequently, increasing the dispersion of nanoparticles reduces the Li migration barrier. However, due to the uneven distribution of sites and the formation of non-uniform Li nuclei, directional Li growth still occurred, resulting in mossy dendrites. Finally, when nanoparticles achieved higher uniformity and density while maintaining the same quantity, uniformly distributed and well-spaced Li nuclei enabled a constant electric field across the electrode, ultimately yielding a dense and smooth deposition morphology (Fig. 4a). Correspondingly, the authors experimentally demonstrated that hosts with densely and uniformly distributed ZnO nanodots enabled more uniform Li nucleation and deposition compared to hosts with dispersed and randomly aggregated morphologies.

In another work by the same group [178], a bottom-up deposition regulation mechanism was established from three aspects nucleation sites, growth patterns, and dead lithium suppression by constructing CuO nanowire arrays with gradient conductivity and lithiophilicity on a copper current collector (denoted as CuO@Cu). In Specific, morphological observations revealed that for sparsely distributed nanowires (S-CuO@Cu), bottom nucleation was achieved. However, due to excessive inter-nanowire spacing, lithium grew upward along isolated nanowires, forming Li clusters that evolved into dendrites. In contrast, densely distributed nanowires (D-CuO@Cu) resulted in an overly compact interface that prevented lithium from accessing the bottom, leading to direct nucleation on the top surface, forming "hot spots" and rapidly inducing dendrite penetration. Only uniformly distributed nanowires (M-CuO@Cu), with moderate spacing and gradient conductivity, enabled lithium to preferentially nucleate at the bottom and subsequently fill internal voids laterally. After stripping, the structure fully recovered without dead lithium residues (Fig. 4b). Furthermore, Liu et al. [179] constructed a conformal 3D Li₁₃Sn₅ alloy framework based on CC (Fig. 4c), which exhibited significant advantages in interfacial stability and dead lithium suppression. From a kinetic perspective, this architecture substantially increased the lithium-ion transference number from 0.24 for bare Li to 0.76. Combined with the uniform electric field distribution and enhanced Li⁺ concentration gradient validated by COMSOL simulations, this enabled rapid ion transport and homogeneous lithium deposition. Moreover, the alloy layer reacted with the electrolyte to form an in situ bilayer SEI structure comprising a Li₁₃Sn₅ inner layer and a LiF outer layer, which ensured high ionic conductivity while providing excellent mechanical strength. Additionally, in situ optical microscopy observations revealed that bare Li formed numerous pits during stripping, which evolved into dendrite networks upon re-deposition. In contrast, the Li₁₃Sn₅@CC electrode exhibited uniform lithium dissolution from the interior and preferential filling of the three-dimensional framework voids. Post-cycling scanning electron microscopy (SEM) confirmed the absence of dendrite and dead lithium accumulation (Fig. 4d), effectively mitigating volume expansion.

In addition, Huo et al. [180] constructed a dynamic self-healing interface (SDI, Fig. 4e) by combining electrospinning and rolling techniques using polyacrylonitrile (PAN) nanofibers and an in situ LiₓSn₅ alloy layer. Herein, the PAN nanofibers, with their polar -C≡N groups, homogenized the Li⁺ flux and served as a reservoir for Sn^2^⁺, while the underlying Li-Sn alloy exhibited a high ionic conductivity of 10⁻^3^–10⁻^4^ S cm⁻^1^. Their synergistic effect effectively reduced the nucleation overpotential. Furthermore, the underlying Li-Sn alloy was uniformly incorporated into the SEI layer, providing continuous and efficient pathways for Li⁺ conduction, thereby enhancing the uniformity of electron/ion distribution at the electrolyte anode interface. Notably, artificial scratch experiments demonstrated that the fractured alloy layer could be repaired in situ within a single cycle. Concurrently, the stable Li⁺ flux guided by the upper polymer nanofiber layer, combined with the stability of the underlying alloy interface, resulted in a smooth and dendrite-free SDI surface after 200 cycles as confirmed by SEM, whereas the bare Li surface was completely covered with dead lithium. These mechanisms ultimately enabled symmetric cells to achieve an ultra-long cycle life exceeding 5200 h with an overpotential of only 16 mV under the harsh conditions of 5 mA cm⁻^2^ and 5 mAh cm⁻^2^.

## Carbon-Based Materials for AFLMBs

### Graphene

**Fig. 5:**
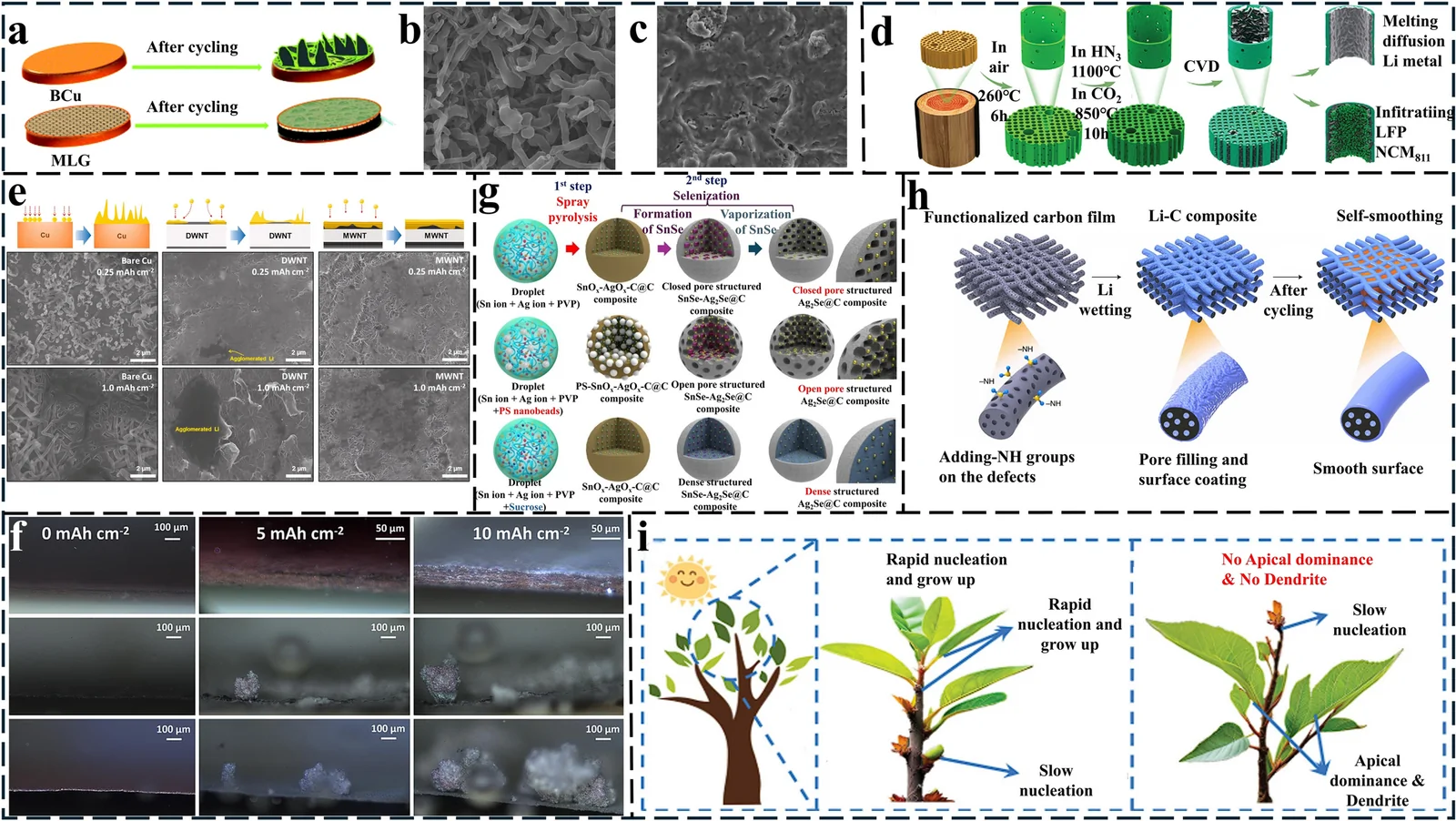
**a** Schematic diagram of Li deposition on bare Cu (upper) and the current collector modified with multilayer graphene (lower). SEM images of **b** bare Cu current collector and **c** the current collector modified with multilayer grapheme [183]. Copyright 2019 Royal Society of Chemistry. **d** Schematic diagram of the preparation process of VGWs@MCF [184]. Copyright 2022 Wiley. **e** Schematic diagrams and SEM images of the effect of bare Cu (left), SWCNT (middle), and MWCNT (right) on Li deposition [121]. Copyright 2022 Elsevier. **f** In situ optical microscopy observations of the lithiation behavior of Cu₂O/Cu@SWCNT (upper), SWCNT (middle), and bare Cu (lower) at a current density of 10 mA cm^−2^ [186]. Copyright 2023, Elsevier. **g** Schematic diagram of the strategy for PC microspheres encapsulating Ag₂Se nanocrystals [190] Copyright. 2025 American Chemical Society. **h** Schematic diagram of the fabrication process for the amine-functionalized mesoporous CF framework [117]. Copyright 2019 Springer Nature. **i** Schematic diagram of the apical dominance mechanism [193]. Copyright 2024 Wiley

Graphene is a honeycomb-like material composed of a single layer of carbon atoms, predominantly exhibiting a layered structure [181, 182]. Possessing exceptional electronic conductivity, mechanical and chemical stability, it endows graphene with widespread applicability in the field of lithium-based batteries. Typical structural variants include multilayer graphene (MLG) and rGO. Via chemical vapor deposition (CVD), the growth of graphene layers can be facilitated on copper foil substrates. During this process, the intrinsic flexibility and mechanical strength of graphene are harnessed to regulate the Li-ion transport flux. In this case, Assegie et al. [183] employed electrochemical polishing technology to obtain ultra-smooth Cu substrates, followed by the growth of graphene films via the CVD process. As a comparison, this modified structure has successfully addressed the issues of “dendritic Li growth” and “instability” in Li deposition on bare Cu (Fig. 5a). SEM images further confirm the distinct differences between graphene-modified and unmodified Cu foils (Fig. 5b, c). While this structure mitigates Li dendrite growth to a certain extent, its 2D architecture suffers from two inherent drawbacks: insufficient spatial accommodation capacity and a lack of lithiophilic sites. These deficiencies lead to inferior lithiophilicity, ultimately resulting in uncontrolled Li dendrite growth. To solve this problem, Mu et al. [184] developed a “dual vertically aligned electrode” design strategy. Based on a 3D multi-channel carbon framework (MCF) substrate, this design incorporates in situ grown 3D hierarchical ZnO and Co_3_O_4_ nanoparticle-anchored vertical graphene nanowalls (denoted as VGWs@MCF) with abundant O/N co-doping (Fig. 5d). Serving as functional units, this structure enables full-dimensional active regulation of Li deposition, thus breaking through the 2D limitation of MLG. In terms of full cell performance, in contrast with MLG/Cu that only optimizes anode, the VGWs@MCF design adopts a dual-electrode vertical alignment, where vertically arranged LiFePO_4_ (LFP) and LiNi_0.8_Co_0.1_Mn_0.1_O_2_ (NCM811) are employed as the cathode to match the VGWs@MCF. This configuration achieves ultra-high mass loading of active materials and low-tortuosity Li⁺ transport pathways, demonstrating high capacity and excellent rate performance. It alleviates the ion transport bottleneck of full cells, realizing dense and dendrite-free Li deposition.

Owing to graphene’s intrinsic excellent electronic conductivity and large SSA, even unmodified graphene demonstrates relatively outstanding Li deposition performance. Furthermore, it is advisable to combine graphene with lithiophilic structures or substances to enhance the uniformity of Li deposition, thereby effectively boosting battery performance. However, graphene has non-negligible drawbacks: its large SSA renders it prone to re-agglomeration/stacking into graphitic structures, and the high cost of fabricating large-scale, defect-free graphene hinders its wider commercial adoption. These issues will be the focus and difficulty of future research in AFLMBs, needing to be further explored.

### Carbon Nanotubes (CNTs)

In contrast with the two-dimensional layered structure of graphene, CNTs feature a unique one-dimensional linear architecture [111, 185]. The unique 1D structure endows them with advantages of lightweight, large SSA, and high flexibility. Leveraging the inherent merits of CFs high mechanical strength, excellent flexibility, and superior electronic conductivity alongside the advantages of 1D material, CNTs show considerable application potential. Kim et al. [121] fabricated and evaluated two CNTs scaffolds with distinct structures, namely double-walled CNTs (DWNTs) and multi-walled CNTs (MWNTs), to explore how the structure of CNTs influences Li deposition behavior (Fig. 5e). The research findings indicate that MWNTs exhibit superior performance compared to DWNTs. The superiority stems from the reversible storage/release of Li⁺ between the walls of MWNTs, which favors uniform and stable Li deposition. Furthermore, the 3D-structured MWNT scaffolds can accommodate excess Li within their void spaces, thereby significantly mitigating volume changes during the drastic migration of Li. While these structural features confirm the architectural advantages of 3D CNT current collectors, two key limitations remain: 1) the unresolved issue of affinity matching between CNTs and Li, 2) uneven Li⁺ concentration distribution in the vertical direction, which causes Li to preferentially deposit on the top surface and fail to fully utilize the internal space of the 3D framework. To tackle this issue, Yang et al. [186] proposed a functionalized design integrating a 3D CNT framework with a lithiophilic gradient heterojunction using SWCNTs as the 3D conductive scaffold and incorporating top-down gradient-distributed Cu₂O/Cu heterojunctions. The merit of this architecture lies in the gradual increase in the density of lithiophilic gradient sites from the top to the bottom, forming a “Li⁺ concentration gradient compensation” effect. This guides Li⁺ deposition from the bottom upwards (instead of surface accumulation), fully utilizing the 3D pores of SWCNTs. In situ optical microscopy observations reveal a minimal volume expansion rate (Fig. 5f). Furthermore, the structure demonstrates ultra-low polarization, rapid kinetics, and excellent full cell performance, addressing the vertical polarization and lithiophilicity issues identified in the Kim group’s work.

Owing to their interconnected 1D network structure, CNTs endow ultra-fast electron transfer kinetics with the incorporation of a certain amount into composites sufficient to form conductive networks. Thus, CNT-based AFLMB anodes exhibit excellent performance under high current densities. In addition, tailoring the structure of CNTs is crucial for suppressing Li dendrite growth. It is proposed to introduce defects or vacancies on CNT surface to anchor lithiophilic species, thereby reducing the Li⁺ nucleation barrier. Like graphene, the network structure of CNTs renders them prone to cross-linking and entanglement, leading to agglomeration. Besides, achieving uniform dispersion of CNTs in composites remains a critical challenge to address.

### Porous Carbon (PC)

Unlike graphene and CNTs, PC materials typically present 3D structures. This architecture possesses inherent merits including a large SSA, low cost, and tunable microstructure alleviating volume fluctuations of electrodes during cycling, homogenizing current density distribution, and facilitating uniform Li deposition [187, 188]. For instance, Zhang et al. [189] developed a structure consisting of a 3D PC framework homogeneously functionalized with sulfur-mediated ultrafine tin clusters. Predominantly mesoporous, this 3D PC framework features excellent pore connectivity facilitating rapid Li⁺ diffusion, and its internal cavities can directly accommodate volume expansion during Li deposition. The pouch cell exhibits a mere 6.6% volume expansion rate after 100 h of cycling, preventing electrode structural collapse. In another study, Seo et al. [190] designed spherical PC materials, in which PC microspheres were used as the shell to encapsulate lithiophilic Ag₂Se nanocrystals internally (Fig. 5g). The merit of this configuration lies in the closed internal pores acting as isolated cavities, confining Li deposition within the pores and preventing Li dendrites from growing toward the surface to contact the electrolyte. Furthermore, it can fully accommodate volume expansion during the repeated Li deposit/stripping, while the rigid carbon shell of the 3D carbon microspheres resists the mechanical stress induced by internal Li deposition, avoiding microsphere collapse. The highly lithiophilic Ag_2_Se within the structure reacts with Li during charge–discharge cycles to form Li-Ag alloys, which provide lithiophilic sites to guide uniform Li nucleation. Meanwhile, the generated Li₂Se serves as an ion-conducting phase to accelerate Li⁺ diffusion inside the pores, addressing the long-standing issue of “slow ion conduction” suffered by conventional carbon-based materials. The symmetric cell based on this structure achieves a voltage polarization of only 20 mV at 3 mA cm⁻^2^.

Benefiting from the intrinsic tunable pore size distribution and ultra-high SSA of PC, it furnishes ample space for ion transport and Li deposition. Additionally, it can be scalably fabricated using active biomass and polymer matrices as precursors. Nevertheless, PC also faces certain challenges: In comparison with graphene and CNTs, it exhibits considerably lower electronic conductivity and inferior mechanical properties when utilized alone as a carbon-based substrate for LMA. This necessitates the incorporation of other composite materials to enhance conductivity and mechanical performance, thereby reducing the lithium nucleation energy barrier and preventing electrode structural collapse.

### Carbon Fibers (CFs)

CF is typically 1D material, with diameters ranging from nanoscale to microscale and structures varying from solid to hollow. CF is another promising host material for AFLMBs due to its high mechanical strength, light weight, excellent flexibility, and superior electronic conductivity [119, 191]. For instance, Niu et al. [117] adopted electrospinning to engineer a stable LMA host based on an amine-functionalized mesoporous CF framework (Fig. 5h). The mesoporous design of this architecture affords ample space for Li deposition, minimizing the accumulation of dead Li. Furthermore, the 3D interconnected structure of CFs homogenizes local current density, avoiding localized Li deposition caused by excessively high local current density. Additionally, via ammonia solution immersion, the defect sites on the CFs surface are amine (-NH) functionalized, which availably boosts the material’s lithium affinity, effectively suppressing Li dendrite formation and growth.

Based on CFs, CC, a 2D structural material consisting of taking CFs as its foundational material, retains the inherent properties of CFs, making it a good candidate host for AFLMBs. However, the intrinsic lithiophobicity of commercial CC results in excessively high Li nucleation overpotential, leading to uneven Li deposition. In hence, several research teams have developed lithiophilic structures based on CC substrates. For example, Yu et al. [192] uniformly deposited silver nanowires (AgNWs) onto the surface of CC fibers via vacuum filtration, constructing an AgNW/CC composite host. In this structure, the CC serves as a 3D porous framework to homogenize local current density, while the deposited AgNWs form Li-Ag alloys with Li by virtue of their high binding energy, augmenting lithiophilic sites to guide uniform Li deposition and reduce nucleation overpotential. The CE remained at 99% in half-cell test. While this design exhibits high CE, the Li⁺ transport rate tends to become unbalanced when the current density increases or the cathode mass loading rises, resulting in preferential Li⁺ deposition on the top of CFs and inducing the “tip effect.” To address this issue, Liu et al. [193] introduced the concept of a “ferromagnetic built-in magnetic field” based on 3D CC and lithiophilic sites. They fabricated a lithiophilic CC@CoF_2_/C composite host via the in situ growth of Co-metal–organic frameworks (MOF) nanosheets on the CC surface, followed by fluoride treatment to form ferromagnetic CoF_2_ nanoparticles. The merit of this structure lies in the Lorentz force generated by the interaction between Li⁺ (moving toward CC@CoF_2_/C under electric field driving) and the built-in magnetic field. This forces Li⁺ deviating from the “top deposition” path and redirect toward the fiber sides and internal pores. The mechanism is analogous to the removal of “apical dominance” in plants (Fig. 5i). The full cell assembled with LFP cathode achieved a high-capacity retention (91.25%) and high CE (99.85%) after 1000 cycles. As a macroscale self-supporting material woven from CFs, CC can directly replace Cu foil as the anode host for AFLMBs. It eliminates the need for active material coating, significantly simplifying the anode fabrication process. Leveraging its intrinsic fibrous structure, CC provides abundant Li deposition sites. However, CC inherently features a low SSA, requiring activation via acidic substances or plasma treatment to introduce abundant defects. Furthermore, enhancing lithiophilicity solely through CC activation is insufficient. Combining with the coating of lithiophilic species on CC is recommended to synergistically improve its lithiophilicity. This is anticipated to become a mainstream approach for suppressing dendrite growth in AFLMBs based on modified CC hosts.

### Li_x_C_y_ Carbides Formation

**Fig. 6:**
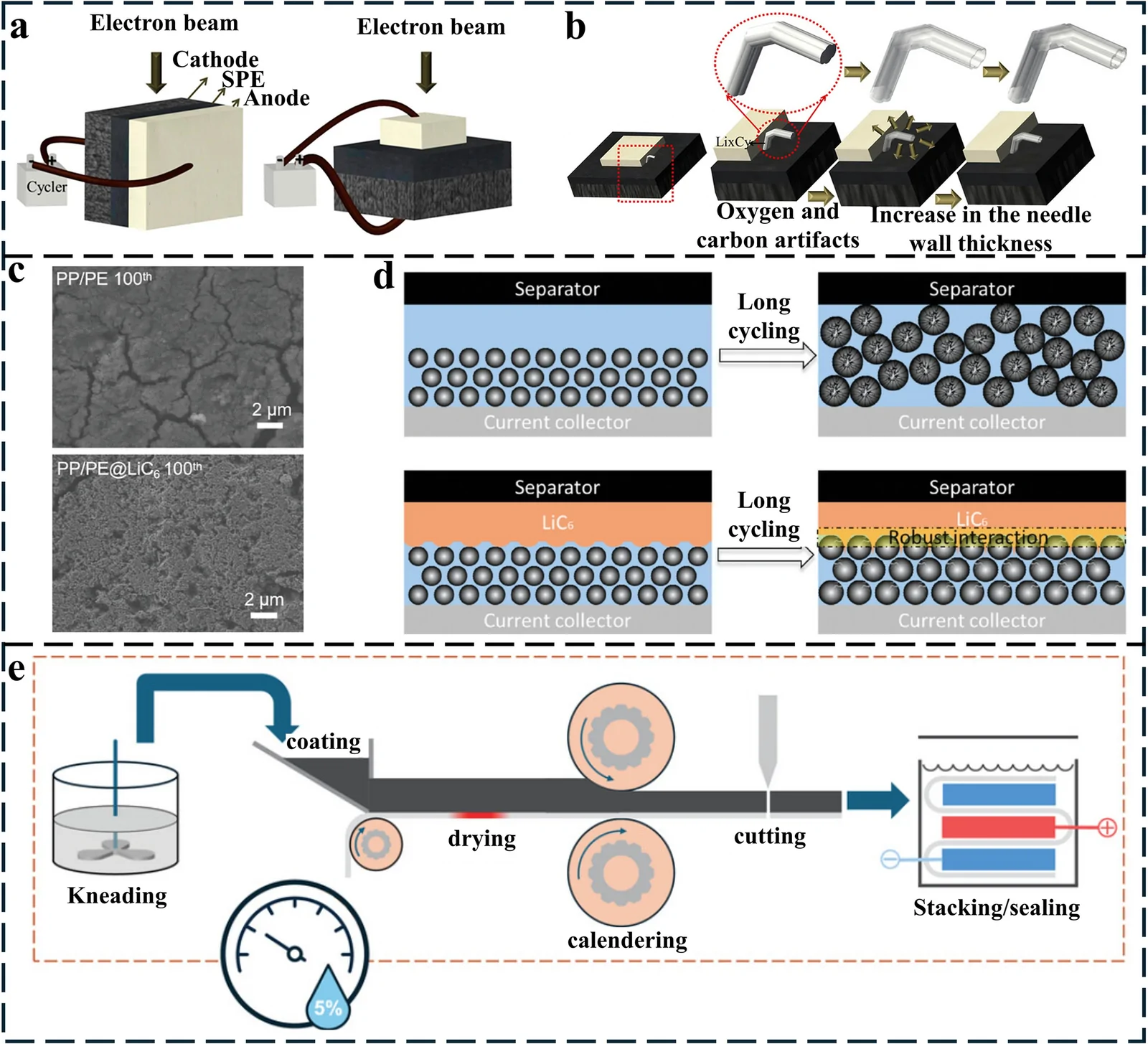
**a** Schematic of the (left) cross-sectional view and (right) plane view of the batteries during in situ experiments.** b** Schematic showing formation of hollow morphology [195]. Copyright 2018 American Chemical Society.** c** Top-view SEM images of Si anode for the Si|PP/PE|Li (up) and Si|PP/PE@LiC_6_|Li (bottom) cells after 100 cycles. **d** Schematic illustrations depict the evolutionary process of Si anode, both in the absence c) and presence d) of a LiC6 coating layer on the PP/PE separator, respectively [196]. Copyright 2025 Wiley. **e** Schematic illustration about the tolerance of Li_2_C_2_ to the Li-ion battery production environment [197]. Copyright 2025 Wiley

It is important to note that when using carbon-based materials as hosts for lithium metal, the chemical interaction between lithium and carbon can lead to the formation of lithium carbides (LiC_x_). This phenomenon has been confirmed in several studies, which demonstrate that lithium can react with the carbon lattice under certain conditions [194, 195]. While the formation of LiC_x_ is often considered an undesirable side reaction that consumes active lithium, recent research suggests that it may also alter the interfacial transport properties [196]. Therefore, a comprehensive understanding of the carbon-lithium interface is essential to either mitigate the formation of detrimental carbides or utilize them constructively to enhance lithiophilicity. For instance, Maryam et al. [195] assembled batteries using a polyether-based solid polymer electrolyte (SPE) with LiTFSI as the lithium salt and LFP as the cathode (Fig. 6a). It was found that carbon-rich species derived from the electrochemical reductive decomposition of SPE react with deposited lithium generated during electrochemical reactions to form Li_x_C_y_. This compound exists not only on the surface but also inside lithium dendrites, and is also a critical component of the SEI. Besides, inhomogeneous SEI (e.g., defects, thin SEI layers) induce uneven lithium deposition, which further promotes the formation of Li_x_C_y_. Meanwhile, under unpressurized or low-pressure conditions (e.g., anode cracks, interfacial gaps), Li can be extruded to form Li_x_C_y_. The formation mechanism is fundamentally attributed to Li plating and stripping during battery cycling, rather than a simple chemical reaction. The presence of Li_x_C_y_ is the key reason why lithium dendrites exhibit higher hardness than bulk lithium metal, enabling such dendrites to penetrate the polymer electrolyte. Subsequently, Li_x_C_y_ further reacts with impurities such as O and C and participates in the evolution of hollow dendrites (Fig. 6b). Moreover, Li_x_C_y_ lacks plastic deformability and thus tends to undergo brittle fractures during battery cycling. The fractured dendrites detach from the anode to form dead lithium, resulting in irreversible lithium loss.

Interestingly, the effects of Li_x_C_y_ on battery performance are not entirely detrimental. Li et al. [196] applied a LiC_6_ coating on the separator to mitigate the expansion of the silicon-based anode. It was found that without the coating, the silicon electrode undergoes expansion, leading to cracking at the electrode interface and accumulation of dead silicon on the Cu current collector. Meanwhile, excessive SEI growth hinders electron transfer between silicon particles, resulting in a decrease in battery capacity. In the case of using LiC_6_ as the coating, a relatively intact electrode structure was exhibited after 100 cycles (Fig. 6c). This is attributed to the strong interaction between the Si anode and LiC_6_ during cycling, which significantly reduces the contact between the electrolyte and Si, effectively restricts the volume expansion of Si particles, and minimizes electrode cracking and delamination. At the same time, the porous structure of LiC_6_ can homogenize the Li^+^ flux, dissipate the shear stress generated by silicon lithiation, retain the intact structure of the electrode, and avoid the deactivation of active silicon (Fig. 6d). From the above discussion, it can be concluded that whether the effect of Li_x_C_y_ is beneficial or detrimental depends on its existing form. For example, when Li_x_C_y_ is loaded on the separator, it can effectively exert its structural characteristics and stabilize the electrode interface. In contrast, when Li_x_C_y_ exists in dendrites, its fragile mechanical properties will damage battery performance and safety. Therefore, focusing on their application scenarios and regulation methods is the key to realizing the positive utilization of Li_x_C_y_.

Notably, Li_x_C_y_ can also be intentionally introduced as a cathode prelithiation agent to compensate for lithium loss in anode-free batteries. Tang et al. [197] demonstrated that Li₂C₂, synthesized via a solid-state reaction, exhibits a high delithiation capacity of 1298.4 mAh g⁻^1^ and stability in dry air (Fig. 6e), enabling its use as an effective prelithiation additive. When incorporated into LNMO or LCO cathodes, Li₂C₂ compensates for irreversible lithium loss during cycling, extending the lifespan of anode-free pouch cells while achieving high energy density (424.1 Wh kg⁻^1^) and power output (1270 W kg⁻^1^). Operando X-ray diffraction (XRD) confirmed that Li₂C₂ decomposition provides additional lithium ions, mitigating capacity fade. This highlights that the controlled application of Li_x_C_y_, such as in prelithiation, can harness its high donor capacity to enhance battery performance, underscoring the importance of tailoring Li_x_C_y_ utilization to specific roles within the battery.

Combined with the basic research theory of the graphite-lithium system and the actual cycling scenarios of lithium batteries, strategies can be formulated to reduce the generation of unintended Li_x_C_y_ and its enrichment at dendrites by regulating temperature, pressure, material interfaces, and actual cycling conditions. Avdeev et al. [194] found that Li_x_C_y_ is mainly formed by the conversion of graphite intercalation compounds (Li-GICs) into Li_x_C_y_ under high-temperature conditions. When the temperature reaches 1000 K, LiC_12_ and LiC_6_ are converted into Li_2_C_2_ due to repeated cooling–heating cycles. Meanwhile, increasing the reaction pressure can raise the conversion temperature. Based on the above research, specific strategies to address the formation of Li_x_C_y_ are proposed as follows: 1) During battery cycling, the operating temperature should be controlled within a safe range, and repeated high-temperature cooling–heating cycles should be avoided to prevent local high temperatures from exceeding the threshold for Li_x_C_y_ formation; 2) apply uniform stacking pressure to the lithium battery system to optimize the contact between the electrode, electrolyte, and separator, and avoid the generation of cracks. At the same time, the temperature threshold for Li_x_C_y_ formation can be increased by raising the pressure; 3) add surface modifiers to the electrode to avoid direct contact and reaction between carbon sources and lithium.

Based on the above overview of Li_x_C_y_, the future research will mainly focus on the development of in situ observation technologies for AFLMBs. Existing studies mainly realize the preliminary characterization of Li_x_C_y_ through in situ SEM and energy-dispersive X-ray spectroscopy (EDS). In the future, it is necessary to develop in situ characterization technologies with higher resolution to realize real-time monitoring of the phase identification, morphological evolution, and composition distribution of Li_x_C_y_ during battery cycling, and clarify its formation and growth rules at the micro-nano scale. Also, it is important to balance the advantages and disadvantages of Li_x_C_y_. For beneficial phases (i.e., LiC₆), it is beneficial to regulate its phase transition temperature through means such as doping and interface coating to prevent its conversion into Li_2_C_2_, avoiding structural fragmentation caused by phase transition during battery cycling and the consequent performance degradation.

To this end, we have systematically summarized four typical carbon-based materials suitable for AFLMBs, namely graphene, CNTs, PC, and CFs. Table 1 compares the advantages and disadvantages of various carbon-based materials used in AFLMBs. Inadequate uniformity of Li deposition on conventional current collectors easily induces Li dendrite growth and dead Li accumulation. Fortunately, these issues can be effectively alleviated when Li is deposited on a substrate with high porosity, high conductivity and larger SSA. The afore-discussed carbon-based hosts address the lithiophobicity and poor volume elasticity of conventional Cu foil current collectors to an extent. Nevertheless, several critical aspects must be emphasized when these materials are utilized: 1) Comprehensive consideration of these materials should be focused on practical application requirements. Ideally, carbon-based hosts with low cost and easy processing, high SSA, light weight, high mechanical strength and excellent conductivity, are prioritized. Compared to non-conductive substrates, conductive ones tend to exhibit lower interfacial impedance and superior electrochemical performance under high current densities, 2) carbon-based materials without any modification possess poor intrinsic lithium affinity, failing to meet the requirements for long cycle life under higher current densities and high Li deposition area capacities. Lithiophilic modification, including surface heteroatom doping, surface decorating, lithiophilic structure design, and lithiophilic gradient design, is considered as an indispensable and effective strategy to enhance the lithiophilic of these carbon-based substrates, which will be elucidated in the following section.

**Table 1:** Comparison of advantages and disadvantages of various carbon materials

Carbon host	Advantages	Disadvantages	References
Graphene	High electrical conductivity, high SSA, and good chemical stability	Single pore structure, prone to agglomeration, high cost, and poor lithiophilicity	[181, 182]
CNTs	High electrical conductivity, high mechanical strength, and a hollow structure	High cost, prone to agglomeration, limited SSA, and lithiophobic	[111, 185]
CFs	High aspect ratio, good mass transfer performance, and a tunable surface	Uneven diameter distribution, low microporosity, structural defects, and lithiophobic	[119, 191]
PC	Ultra-high SSA, tunable pore structure, and strong adsorption capacity	Poor electrical conductivity, structural instability, high cost, and poor lithiophilicity	[187, 188]

## Lithiophilic Modifications for Carbon-Based Hosts

### Surface Heteroatom Doping

**Fig. 7:**
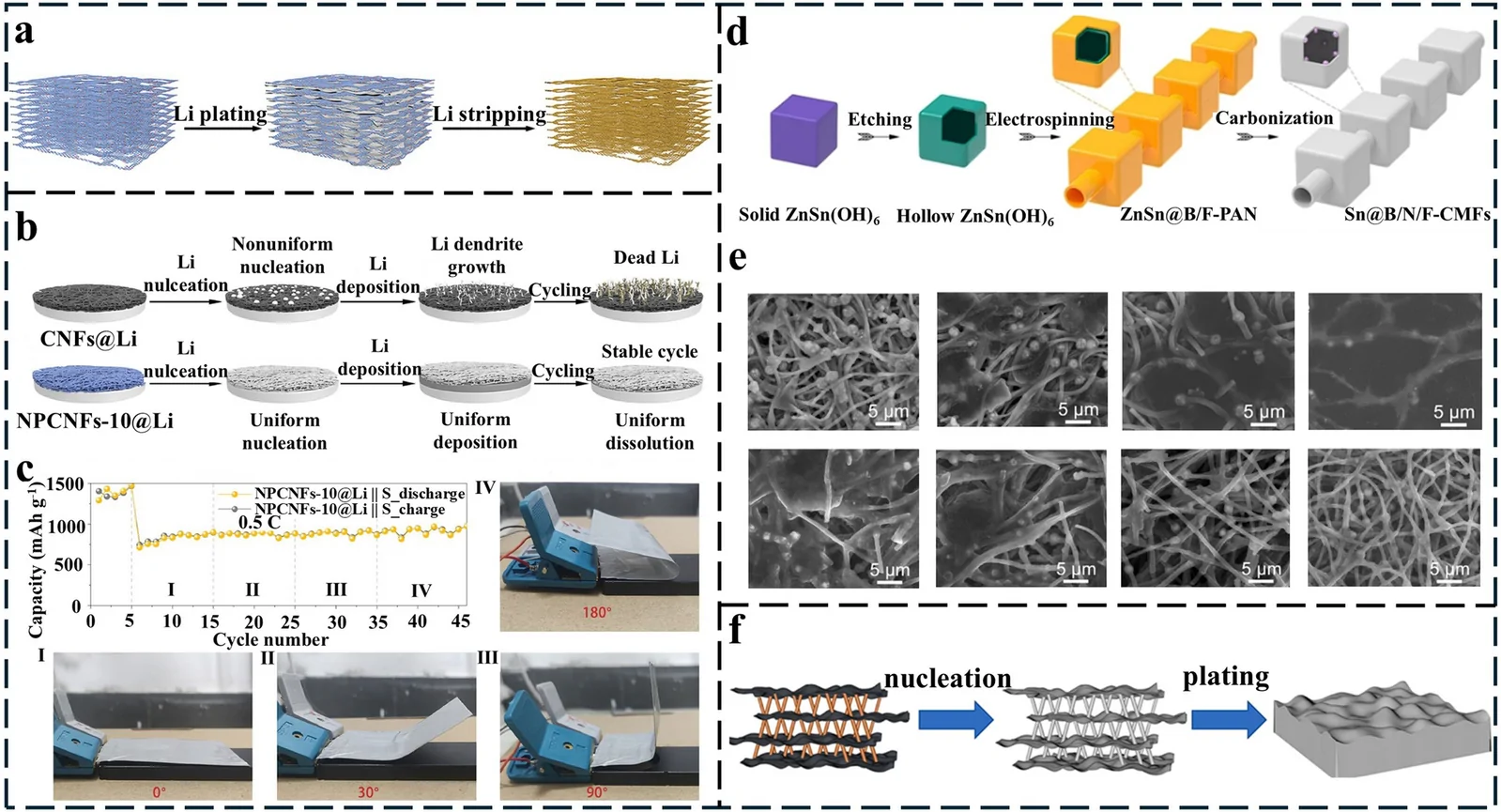
**a** Schematic diagram of Li deposition/stripping on NGM [202]. Copyright 2023 Wiley. **b** Schematic diagrams of Li deposition/stripping on bare CNFs (upper) and NPCNFs (lower). **c** Performance of full cells assembled with NPCNFs and S cathode under different bending angles [203]. Copyright 2024 Elsevier. **d** Schematic diagram of the preparation process of Sn@B/N/F-CMF. **e** SEM images of Li deposition (upper) and stripping (lower) on the necklace-like structure of Sn@B/N/F-CMF [204]. Copyright 2025 Wiley. **f** Schematic diagram of the Li deposition/stripping mechanism on S/N-rGO/MWCNTs [205]. Copyright 2025 Elsevier

Boosting the surface lithiophilicity of carbon-based materials can effectively reduce the initial nucleation barrier during Li deposition, and it is one effective approach to strengthen the interaction between Li and carbon-based materials [198, 199]. Doping electronegative elements (e.g., N, P, B, F, and S) with Li-interactive orbitals onto the surface of carbon-based materials enables the enhancement of their lithiophilicity. For instance, N-doped graphene has been employed as a functional coating on the lithium metal surface. It has been found that pyridinic N and pyrrolic N in N-doped graphene are lithiophilic and can facilitate the uniform distribution of Li. This is attributed to the fact that pyridinic N contains a lone pair of electrons, while pyrrolic N possesses one extra electron; both act as electron-rich sites (Lewis bases). They can strongly adsorb Lewis acidic Li⁺ via acid–base interactions, providing uniform anchoring sites for Li nucleation [200, 201]. This finding confirms that N is lithiophilic; accordingly, several research groups have developed nitrogen-doped graphene networks (NGM) [202]. In this structure, the abundant nanopores not only afford ample space for Li⁺ deposition (Fig. 7a), but also facilitate rapid Li⁺ transport, enabling high rate performance. Firstly, the nanopores shorten Li⁺ migration pathways while the interlayer channels accommodate Li deposition. Meanwhile, the large SSA homogenizes local current density, preventing local overcurrent-induced Li dendrite growth. Second, leveraging the lithiophilicity of N atoms, uniform nucleation sites are constructed to guide the ordered deposition of Li, thereby suppressing dendrite growth. Finally, the high flexibility of the graphene-based substrate further accommodates volume changes induced by Li deposition/stripping during cycling, avoiding electrode structural rupture.

While the single-atom doping of this structure delivers improved performance, it only contains a single type of N atom, thus affording limited regulation over lithiophilicity. To breakthrough this limitation, Du et al. [203] devised N, P co-doped PC nanofibers (NPCNFs), incorporating P atoms alongside N doping, further lowering the Li⁺ nucleation barrier. Meanwhile, structural advancements were also achieved: advancing from 2D lamellar stacking to 3D fibrous interweaving, which enhances the continuity of the fiber network, and transforming from disordered pores to oriented channels. This unique configuration not only ensures rapid ion transport but also dissipates the stress induced by Li deposition via the channels (Fig. 7b). The nucleation overpotential decreased from 19 mV exhibited by NGM to 14 mV for NPCNFs, demonstrating that dual doping outperforms single doping in enhancing lithiophilicity and promoting more uniform Li Nucleation. Furthermore, a flexible pouch full cell was assembled using NPCNFs as anode host pairing an S cathode. Under bending conditions ranging from 0° to 180°, it retained a capacity retention rate of 71.7% after 90 cycles at 0.5 C (Fig. 7c), validating the structural stability of the configuration.

Beyond single-atom and dual-atom doping, several groups have developed tri-atom doping strategies. For instance, Tian et al. [204] engineered a lightweight and functionalized scaffold composed of Sn nanoparticles embedded in necklace-like B, N, F-doped carbon macroporous fibers (Sn@B/N/F-CMFs) (Fig. 7d). The three lithiophilic elements (B, N, F) are uniformly doped into the carbon framework via carbonization, achieving full area lithiophilic coverage on both the inner and outer surfaces of the carbon shell. This enhances Li⁺ adsorption capacity and suppresses Li dendrite growth induced by the tip effect. Moreover, the necklace-like 3D interconnected fibrous structure of Sn@B/N/F-CMFs forms a conductive pathway spanning the entire electrode, effectively homogenizing local current density. Additionally, B, N, F co-doping imparts enhanced flexibility to the carbon-based substrate, enabling the necklace-like fibers to withstand repeated deformation and realize reversible Li deposition and stripping on the structure (Fig. 7e), thus preventing overall structural collapse caused by local expansion. When assembled into an AFLMB with a 7.5 mg cm⁻^2^ high-loading 5 V-grade LiNi_0.5_Mn_1.5_O_4_ (LNMO) cathode, it retains a high-capacity retention of 90% after 160 cycles.

Besides N, P, and B doping, other atomic doping strategies, such as S and F doping, also prove effectiveness in boosting lithiophilicity. For instance, the Li’s group [206] developed laser-induced fluorine-doped graphene arrays (F-LIG-A), optimizing Li adsorption and nucleation behaviors at the atomic level. Density functional theory (DFT) simulations reveal that unlike pristine graphene which induces vertical Li growth on its surface, this architecture guides Li to spread laterally along the graphene plane, suppressing Li dendrite growth caused by the tip effect in the vertical direction. Furthermore, as F is a key component of the SEI, fluorine doping in F-LIG-A not only optimizes Li nucleation but also reacts in situ with Li to form a LiF-rich SEI featuring high mechanical strength and excellent ionic conductivity. This addresses the issues of poor mechanical stability and excessive side reactions in conventional SEI. When assembled into a full cell with a high-loading LFP cathode (20 mg cm⁻^2^), it retains a capacity retention of 82.0% after 80 cycles at 0.5 C. Also, Tong et al. [205] fabricated a graphene carbon framework co-doped with S and N, integrated with MWCNTs (denoted as S/N-rGO/MWCNTs). S/N co-doping remarkably enhances the lithiophilicity of the graphene-based carbon matrix, thereby facilitating the uniform deposition of metallic Li. Meanwhile, the graphene framework modulates the local current density on the electrode to suppress Li dendrite growth (Fig. 7f). Integrating these structural merits, the S/N-rGO/MWCNT composite anode exhibits an outstanding charge–discharge performance. At a current density of 1 mA cm⁻^2^ and an area capacity of 1 mAh cm⁻^2^, it achieves a CE of 96.8% after 550 cycles. The composite anode maintains a CE of 94.9% after 500 cycles even at a high current density of 3 mA cm⁻^2^. With minimal CE degradation, the composite anode demonstrates exceptional electrochemical performance.

In summary, this section systematically summarizes the surface heteroatom doping engineering of carbon-based hosts for AFLMBs, mainly encompassing single-atom, dual-atom and multi-atom doping. Atomic doping targetedly addresses the long-standing challenges of poor lithiophilicity, SEI instability, and Li dendrite growth. The underlying mechanism lies in the fact that these doped atoms react with Li⁺ to form ion-conductive composite layers (e.g., Li_3_N, LiF, Li_2_S), which construct a high-conductivity network on the 3D carbon-based substrate. This synergistically enhances electrical conductivity and lithiophilicity, leading to a uniform Li deposition. Meanwhile, these substances incorporate into the SEI layer to enhance its mechanical robustness and supplement its inorganic components, mitigating the continuous consumption of active Li resulted from repeated SEI rupture/reconstruction, a key factor contributing to low CE. Despite the promising prospects of heteroatom doping, it still faces certain limitations: 1) Current doping processes lack validation of performance under harsh conditions, such as high current densities, high mass loadings, and low negative/positive (N/P) capacity ratios, 2) atomic-level uniform distribution is challenging, to which, both excessive and insufficient doping can induce Li dendrite growth, 3) the mechanism of multi-element co-doping remains unclear, hindering the design of optimal doping combinations. Thus, it is necessary to introduce highly lithiophilic species to decorate carbon-based hosts to reduce the reliance on heteroatom doping strategies.

### Surface Decorating

**Fig. 8:**
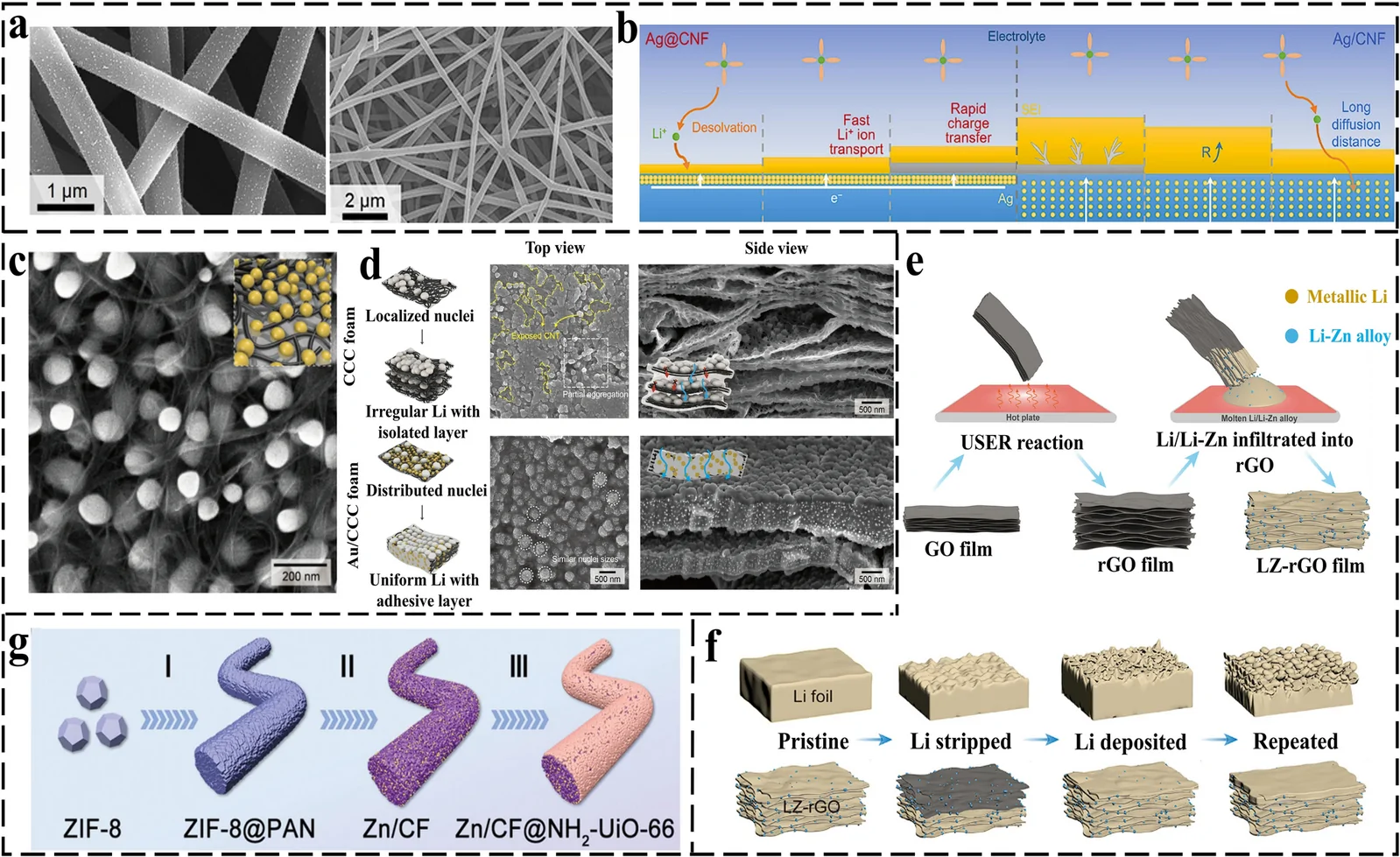
**a** SEM images of Ag@CNT (left) and Ag/CNT (right). **b** Schematic diagram of SEI formation and Li deposition on Ag@CNT [211]. Copyright 2025 Wiley. **c** High-resolution SEM images of Au/CCC. **d** Li deposition behaviors of CCC (upper) and Au/CCC (lower), and SEM images of top-view and side-view after depositing 1.0 mAh cm⁻^2^ [107]. Copyright 2023 Royal Society of Chemistry. **e** Schematic diagram of the fabrication process of the LZ-rGO structure. **f** Schematic diagrams of the Li deposition/stripping process on Li foil (upper) and LZ-rGO (lower) [212]. Copyright 2024 Wiley. **g** Schematic diagram of the preparation route of Zn/CF@NH_2_-UiO-66 [213]. Copyright 2025 Wiley

Stable and reversible deposition/stripping of metallic Li is of great significance to AFLMBs. Accordingly, surface decorating strategies for carbon-based hosts were further summarized. Surface alloying, as one effective surface decorating approach, is the most commonly used method to enhance the lithium affinity of carbon hosts. Compared to surface heteroatom doping, alloying is more conducive to reduce lithium nucleation overpotential and render lithium nucleation sites more uniform [207–209]. This theory was earlier validated by researchers. For example, the Cui’s group [210] demonstrated that various metals can react with Li to form Li-M alloys (M = Ag, Au, Zn, Mg, etc.) by utilizing their binary phase diagrams with Li. These Li-M alloys exhibit a certain solubility of Li metal, acting as buffer layers to reduce the Li nucleation barrier. Among them, Ag stands as the most representative one, holding the broadest application prospects in both LMBs and AFLMBs. However, the loading capacity of silver nanoparticle (AgNPs) on carbon-based hosts is usually insufficient, especially in the commonly used conventional electrospinning technology. To address the limitation, Peng et al. [211] developed a carbon nanofiber (CNF) scaffold with controllably anchored AgNPs on its surface (Ag@CNF) via electrospinning combined with ion exchange strategy. Different from the conventional AgNPs-loaded CNFs (Ag/CNF) prepared by electrospinning alone, this strategy markedly enhances the AgNPs loading capacity, which is clearly reflected in the significant difference in loading density between the two samples in the SEM images (Fig. 8a). Notably, the advantage of this structural design is attributable to the exposure of active sites. In contrast with conventional Ag/CNF host where active sites are encapsulated within the CNF matrix, this architecture shortens Li⁺ diffusion pathways, reduces the nucleation overpotential, and facilitates uniform Li deposition inside the structure. Furthermore, Ag@CNF further enables a thinner SEI layer and lower charge transfer resistance, attributing to its superior structural features that reflect the synergistic effect of reduced interfacial energy and regulated electronic states (Fig. 8b). And finally, the LFP//Ag@CNF full cell retains 90% of its initial capacity after 300 cycles, under the conditions of an area capacity of 1.5 mAh cm⁻^2^ and low N/P ratio of 2.0.

Beyond Ag-based alloys, Au-based alloys also exhibit considerable advantages in guiding uniform Li deposition. For instance, the Jung’s group [107] employed a nanocellulose-assisted carbon thermal shock (CTS) method to anchor ultra-high mass fraction (65.0 wt%) Au nanoparticle seeds (AuNPs) on a CNT foam (denoted as Au/CCC), in which, the AuNPs are uniformly dispersed and embedded within the CNT foam (Fig. 8c). Benefiting from the formation of an adhesive interlayer induced by the high-loading AuNPs, this architecture effectively mitigates volume expansion within the electrode and suppresses Li dendrite growth. Unlike pristine CNT foam, where Li deposition occurs on the surface, the AuNPs in this configuration guide uniform Li deposition, enabling dense Li deposition within the space provided by the CNT foam (Fig. 8d). Furthermore, AuNPs significantly reduce the Li nucleation barrier and remarkably improve reaction kinetics between AuNPs and Li. The strong adhesion between AuNPs and the CNT foam provides robust support for the stability of electrochemical reactions, delivering an ultra-long cycle of 650 cycles in carbonate-based electrolytes. The above works clearly validate the effectiveness in improving the lithiophilicity of carbon hosts and thus suppressing dendritic Li growth through decorating highly lithiophilic metals on carbon hosts.

Apart from metal nanoparticles deposited on carbon substrates wherein they indirectly react with Li to form Li-M alloy solid solutions and reduce the Li nucleation barrier, several groups have explored directly forming Li-M alloy solid solutions combined with carbon substrates as high-performance dendrite-free Li metal anodes. For instance, the Chen’s group [212] synthesized a novel LMA host (denoted as LZ-rGO) via a simple melt-infiltration method by integrating 2D rGO with lithiophilic Li-Zn alloy nanoparticles (Fig. 8e). In this structural design, the high SSA of rGO mitigates local current density and retards Li dendrite growth. Unlike conventional rGO plagued by the long-standing issue of easy aggregation and consequent SSA reduction, the Li-Zn alloy nanoparticles in LZ-rGO are firmly anchored on rGO sheets, acting as structural spacers to inhibit rGO aggregation. Furthermore, the Li deposition/stripping mechanism highlights the remarkable advantages of this architecture. Conventional Li foil, with its limited SSA, restricts Li⁺ to deposit directly on its surface triggering repeated chemical reactions with the electrolyte and consequent uncontrolled dead Li deposit and dendrite growth. In contrast, for LZ-rGO, Li⁺ permeates into the internal electrode structure and accumulates around Li-Zn nanoalloys, synergistically reducing the Li⁺ nucleation overpotential and facilitating uniform Li deposition (Fig. 8f). When paired with an LFP cathode (LZ-rGO||LFP), it achieves exceptional capacity retention of 97.9% after 900 stable cycles at a high rate of 3.0 C, validating the structural superiority.

Besides Au, Ag, and Zn, other metal elements have also exhibited good electrochemical performance. The Yin’s group [127] introduced Co single atoms into N-doped carbon nanosheets (Co/NC) to enhance interactions with Li⁺/Li. Co/NC exhibits optimal chemical softness, yielding the lowest nucleation/growth overpotentials among metal/NC substrates. Symmetric cells ran stably for 657 h (avg. CE 99.2%). Full cells with high-loading NCM811 retained 98.8% capacity after 150 cycles, guiding high-performance AFLMBs development. In addition, researchers have proposed leveraging metal–organic frameworks (MOFs) to enhance ion diffusion kinetics. Benefiting from their intrinsic ordered channels and unique porous structures, MOFs afford ample flexibility for Li⁺ diffusion in the electrolyte and across the separator. For instance, the Yu’s group [213] fabricated a bifunctional mixed ionic/electronic conductor for dendrite-free LMA. This design integrates two MOF structures: zeolitic imidazolate framework-8 (ZIF-8) and the rigid MOF (UiO-66) with zirconium (Zr) as the metal center and terephthalic acid as the organic ligand (Fig. 8g). Notably, after high-temperature treatment of ZIF-8 and PAN, the derived Zn-containing PC fibers afford abundant lithiophilic sites. Subsequently, amine-functionalization of UiO-66 (NH2-UiO-66) serves as the fiber outer layer to accelerate Li⁺ diffusion. Ion migration tests demonstrate that the Li⁺ migration barrier of NH2-UiO-66 (0.085 eV) is significantly lower than that of pristine UiO-66 (0.117 eV), validating its role in promoting Li^+^ diffusion. Symmetric cells achieve stable cycling for 2400 h at 1.0 mA cm⁻^2^, with a smooth and dendrite-free electrode surface after cycling. This is attributed to the SEI-stabilizing effect of NH₂-UiO-66 and the lithiophilicity afforded by Zn alloys. Furthermore, full cell assembled with LFP deliver a high-capacity retention of 93.4% and a CE of up to 99% after 1700 cycles at 2.0 C. These aforementioned works convincingly demonstrate the effectiveness of surface alloying in improving the lithiophilicity of carbon-based hosts.

**Fig. 9:**
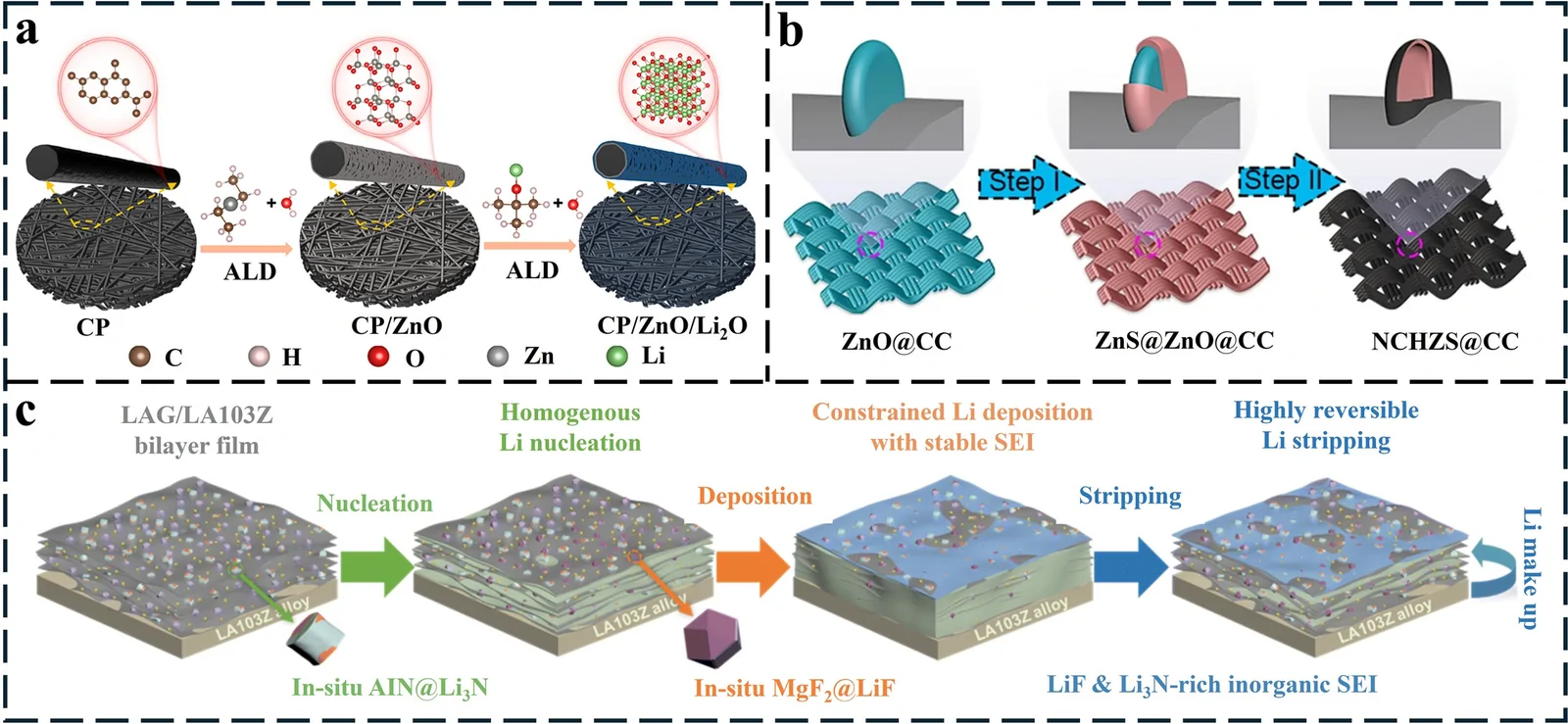
**a** Schematic diagram of the fabrication process of the CP/ZnO/Li₂O current collector [214]. Copyright 2025 Elsevier. **b** Synthetic scheme of NCHZS@CC [215]. Copyright 2025 Springer Nature. **c** Schematic illustrations of the morphological and interfacial evolutions of the LAG film [216]. Copyright 2025 Royal Society of Chemistry

Compared to surface alloying, surface decorating with metal oxides or metal compounds also exhibits distinct Li deposition mechanism. For instance, ZnO serves as a typical example. To further optimize interfacial chemistry and deposition kinetics, the Zhang’s group [214] used atomic layer deposition (ALD) to deposit a ZnO/Li₂O bilayer on carbon paper (CP/ZnO/Li₂O, Fig. 9a). The bottom ZnO provides lithiophilic nucleation sites, while the top Li₂O layer offers electron insulation and ion conduction—inhibiting Li⁺ tunneling, enhancing transport kinetics, and promoting a LiF-rich SEI. Combined with the porous CP substrate, this design improves 3D framework utilization, enables uniform Li deposition and mitigates volume expansion. The design using ZnS as lithiophilic material can also enhance the stability of the SEI and mitigate volume expansion. For instance, Zhou et al. [215] developed a 3D lithiophilic scaffold (NCHZS@CC, Fig. 9b) by integrating ZnS nanosheets with N-doped carbon shells on CC. The interwoven carbon fiber network disperses current density, while the 3D structure buffers volume changes. During cycling, ZnS reacts with Li to form lithiophilic Li-Zn alloys and Li₂S, which stabilizes the SEI. The nanocapsule structure also prevents active material loss. When paired with a high-loading LFP cathode (11.5 mg cm⁻^2^), the full cell retains 86.3% capacity after 900 cycles at 2.0 C.

Beyond Zn-based compounds, other metal compound-decorated carbon architectures have also demonstrated effective regulation of Li deposition. For example, Bai et al. [216] designed a free-standing porous graphene film decorated with lithiophilic AlN@Li_3_N, MgF_2_, and Li₂CO_3_ particles, laminated with a Mg-Li alloy foil (LA103Z) to compensate for Li loss (Fig. 9c). The porous graphene framework accommodates volume expansion and distributes current density, while the inorganic particles play dual roles: AlN@Li_3_N and MgF_2_ guide uniform Li nucleation and promote the in situ formation of a LiF and Li_3_N-rich inorganic SEI during cycling. This robust SEI enhances Li⁺ transport and stabilizes the electrode/electrolyte interface. The integrated LA103Z foil continuously supplies Li to offset irreversible consumption. Consequently, when paired with a high-loading NCM811 cathode, this anode-free full cell achieved a capacity retention of ~ 60% after 120 cycles at 1.93 mA cm⁻^2^ with an average CE of 99.4%, demonstrating the effectiveness of integrating multiple functional components into a carbon host.

In summary, this section summarizes the mechanisms and advances of decorated carbon-based hosts with lithiophilic metals and metal oxides in promoting uniform Li deposition and restraining volume variation. These surface decorating strategies substantially enhance the lithiophilicity, cycling stability and rate capability of carbon-based substrates. Compared to heteroatom doping, surface alloying is more facile to implement on 3D carbon-based substrates unlike the high-temperature calcination required for atomic doping, alloying composite modification can be successfully achieved low-temperature hydrothermal treatment, significantly simplifying the manufacturing process. Notably, beyond the alloys, metal oxides and compounds discussed herein, other alloys and metal oxides (e.g., Mg [108], In [217], Co [218], MgO [219], etc.) also exhibit similar effects. Although such proposals demonstrate remarkable efficacy in improving Li deposition uniformity and suppressing Li dendrite growth, structural design optimization is still required at the electrode level to further promote uniform Li deposition and especially mitigate volume expansion caused by repeated Li deposition/stripping during cycling. Furthermore, an indispensable consideration is the design of more robust growth modes on 3D carbon-based hosts to prevent coating detachment during long-term cycling, which would otherwise degrade battery performance. Additionally, decorating 3D carbon-based materials incorporating bimetallic and trimetallic nanoparticles are currently under exploration. Thus, designing a rational and stable structure is a decisive step to stabilize lithiophilic sites.

### Lithiophilic Structural Engineering and Framework Design

**Fig. 10:**
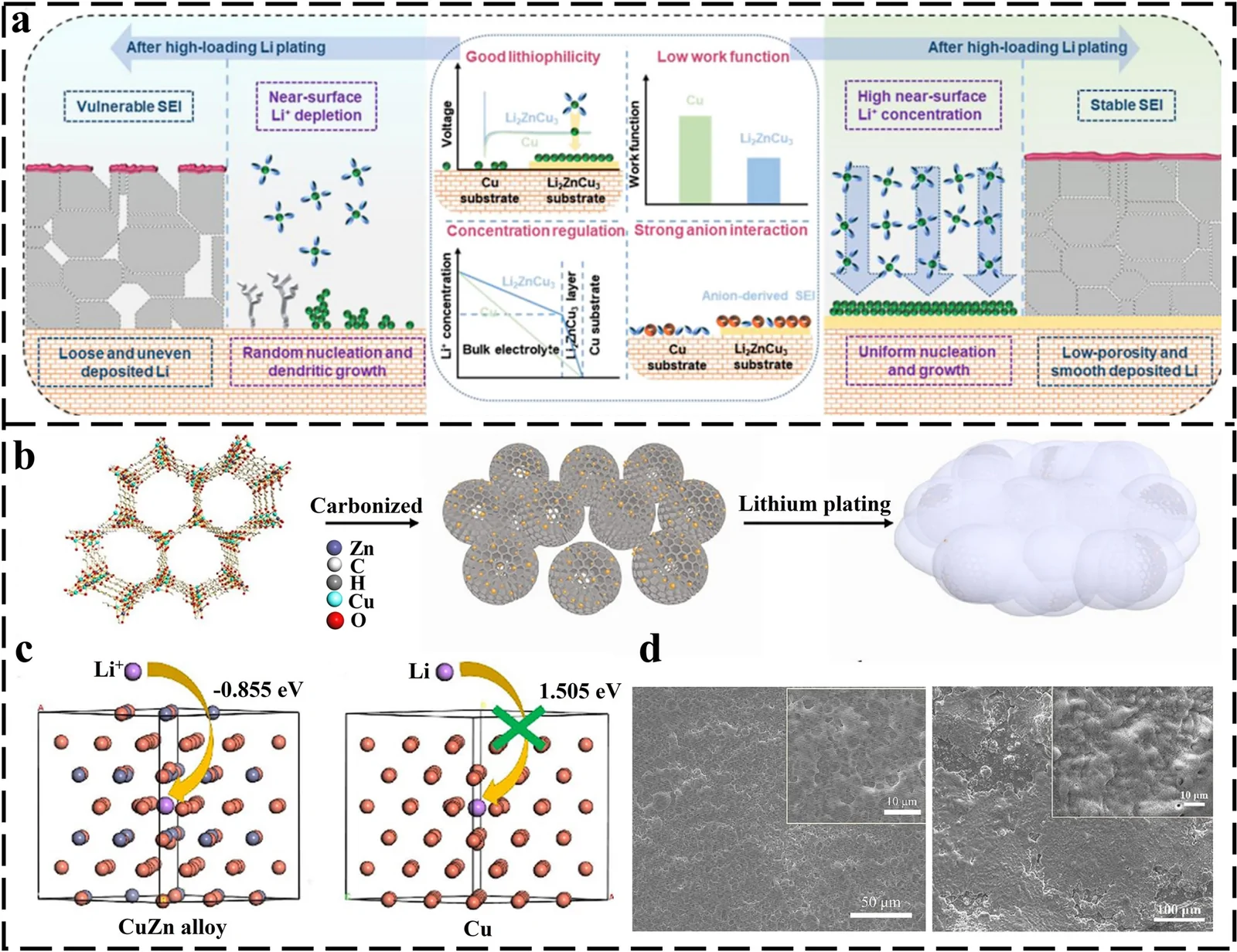
**a** Comparative schematic diagram of Li plating/stripping modes on Cu electrode and Li₂ZnCu₃@Cu electrode [223]. Copyright 2024 Wiley. **b** Schematic diagram of the synthesis process of CuZn MOF. **c** DFT calculation results (left) and CuZn alloy (right), bare Cu. **d** SEM images of the electrode surface of CuZn alloy clusters after 50 cycles (Left), SEM images of the electrode surface of CuZn MOF composite anode after depositing 1 mAh cm^−2^ (Right) [224]. Copyright 2025 Elsevier

In the preceding discussion, we have generally summarized the effects of introducing lithiophilic sites on Li deposition, including strategies such as surface heteroatom doping and surface decorating (surface alloying to form Li metal solid solution). Distinguished from above-discussed surface modification strategies, utilizing the host material as a stabilizing medium to accommodate Li deposition proves a viable strategy to address the challenges associated with uncontrolled volume expansion. With advances in technology, significant progress has been made in designing 3D bulk structures composed of metals, carbon, and polymers [3, 123, 220, 221]. In this section, lithiophilic structural engineering and framework design will be discussed, emphasizing the physical framework construction (e.g., 3D lithiophilic frameworks, hollow and spherical architectures, etc.). To begin with, it is essential to examine the influence of metal substrate modification on Li deposition behavior research in this field has laid a solid foundation for subsequent modifications of carbon-based materials. Lai et al. [222] revealed at the atomic level the influence of different crystal facets of Cu substrates on Li deposition behavior via large-scale molecular dynamic (MD) simulations combined with surface similarity analysis. Studies have shown that the Cu (100) and (111) facets can induce Li to form a stable Li (110) crystal structure on Cu substrates due to their low and uniformly distributed surface potential. In contrast, the Cu (110) facet tends to induce dendrite growth due to its unevenly distributed surface potential. Building on this theoretical foundation, Cao et al. [223] pushed the modification of metal substrates to the stage of multi-metal composite functional enhancement. They engineered a Cu electrode modified with a ternary Li_2_ZnCu_3_ alloy. Electrochemical tests demonstrate that this composite electrode exhibits a surface work function of 3.93 eV significantly lower than that of the Cu (111) facet (4.76 eV) and possesses strong binding affinity with TFSI⁻ anions (Fig. 10a). This design reduces the nucleation overpotential to 106 mV and remarkably enhances the electrode lithiophilicity.

To further enhance the nucleation guidance and durability of Li, Zhu et al. [224] engineered a more advanced architecture with CuZn alloy clusters supported on carbonized MOFs (Fig. 10b). This structure ingeniously integrates the lithiophilicity of the alloy clusters and the spatial confinement capacity for Li deposition of the carbonized framework. DFT calculations reveal that CuZn alloy reduces the binding energy with Li⁺ to -0.885 eV (Fig. 10c), dramatically promoting Li deposition. The composite anode exhibits an ultra-low nucleation overpotential of 8.7 mV. It maintains a uniform, dense, and dendrite-free morphology after charge–discharge cycling (Fig. 10d). And the full cell based on this architecture delivers a high-capacity retention of 90% after 600 cycles. These studies establish the synergistic design concept of lithiophilic sites and structural support, which not only deepens the understanding of lithiophilic mechanisms but also provides crucial insights for subsequent lithiophilic structure designs for carbon-based hosts, inspiring the development of various high-efficiency carbon-based lithiophilic architectures.

**Fig. 11:**
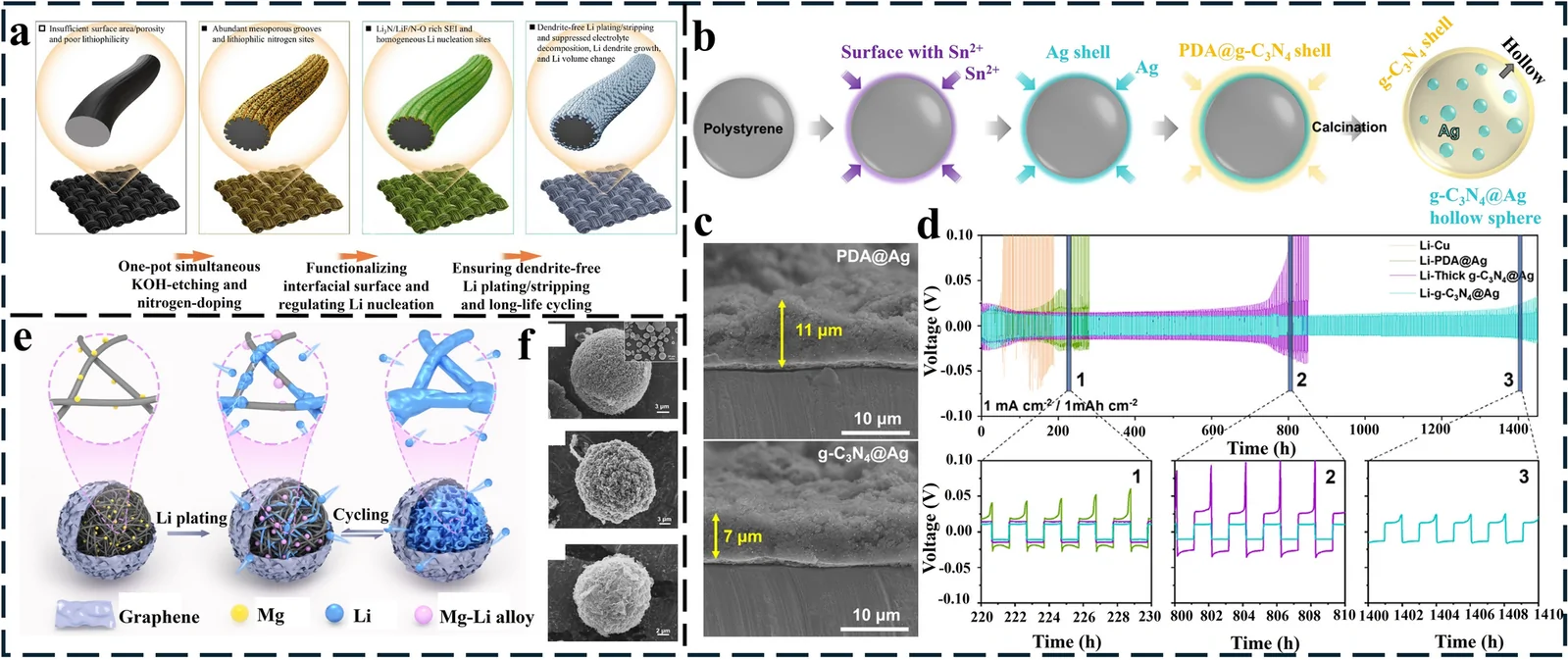
**a** Schematic diagram of the synthesis process of the multifunctional KNCC composite anode [225]. Copyright 2023 Royal Society of Chemistry. **b** Schematic diagram of the synthesis process of g-C_3_N_4_@Ag hollow spheres. **c** SEM cross-sectional image and schematic diagram of the thickness of PDA@Ag during Li deposition (Upper), schematic diagram of the thickness of g-C_3_N_4_ during Li deposition (Lower). **d** Durability schematic diagram of symmetric cells [226]. Copyright 2024 Wiley. **e** Schematic diagram of the structure of the NC/Mg@rGO composite scaffold.** f** SEM images of NCM (top), NC/Mg (middle), and NC/Mg@rGO MS (bottom) [120]. Copyright 2024 Elsevier

Building on the above research on metal substrate (i.e., Cu) modification, the research focus has further shifted to carbon-based materials whose inherent excellent microstructures and physical properties offer an alternative approach for lithiophilic structure design. For instance, it integrates both heteroatom doping and surface etching into 3D carbon-based hosts. This design affords structural support, spatial buffering, and lithiophilic sites, thereby further guiding uniform Li deposition and accommodating volume fluctuation. For example, our previous work [225] conducted simultaneous N doping and KOH etching on the carbon fiber surface of commercial CC via one-step hydrothermal method, increasing the SSA and creating abundant N-containing lithiophilic defect sites to obtain the KOH-etched and N-doped CC (KNCC) multifunctional host (Fig. 11a). The core advantage of this architecture lies in KOH etching-induced SSA enhancement and the improved lithiophilicity from high-content N doping (5.32 at%). The etched mesoporous structure increases the active sites of the CC host, facilitating electrolyte wettability and ion transport, while the doped N (particularly pyrrolic and pyridinic N) further enhances the affinity to Li. When assembled into a full cell with a high-loading LFP cathode (14 mg cm⁻^2^), it delivers a capacity retention of 65.5% at a high rate of 10.0 C, as well as maintaining 86.0% of its initial capacity with an average CE of 96.2% after 500 cycles at 1.0 C, demonstrating that this unique structure design maintains excellent electrochemical performance under high-loading and variable-rate conditions.

To achieve high-performance LMBs or AFLMBs, the design of lithiophilic structures has expanded from 3D porous frameworks to spherical micro-/nanostructures. Their unique structural characteristics such as high SSA and uniform pore distribution facilitate the maintenance of a stable electrode–electrolyte interface during cycling. Lim et al. [226] engineered a unique hollow nanosphere architecture denoted as g-C_3_N_4_@Ag hollow spheres (Fig. 11b), which incorporates Ag nanoparticle seeds into graphitic carbon nitride (g-C_3_N_4_) shells. Consequently, the spherical structure provides tunable internal space that not only encapsulates Li metal to avoid side reactions with the electrolyte, but also mitigates volume changes induced by Li deposition/stripping, preventing electrode structural collapse caused by volume expansion. Besides, the Ag nanoparticle seeds distributed in the inner layer react with Li to form Li-Ag alloys, affording lithiophilic sites and effectively reducing the nucleation barrier (ultra-low nucleation overpotential of 1.28 mV) to precisely guide the oriented growth of Li inside the spheres. Additionally, polydopamine (PDA) is introduced as an adhesive layer on the outer shell, facilitating the uniform coating of g-C_3_N_4_ on Ag nanoparticle layers. Compared to PDA alone, Li deposition on the host surface exhibits a smaller thickness when g-C_3_N_4_ is involved (Fig. 11c). Furthermore, the high conductivity and ionic transport capability of the spherical outer shell largely enhance the suppression of dendrite growth. This synergistic design endows the g-C_3_N_4_@Ag host with extremely stable long-term cycling of 1400 h with a low overpotential of 20.0 mV (Fig. 11d). Finally, the assembled full cell (LiNi_0.90_Co_0.07_Mn_0.03_O_2_||Li-g-C_3_N_4_@Ag) shows a stable cycling performance with a discharge capacity of 138.7 mAh g^−1^ even after 200 cycles (CE over 99% and capacity retention over 67%).

Above, we have summarized the lithiophilic modification and structure designs for 3D carbon-based and spherical structural hosts, which have demonstrated favorable cycling performance. To achieve superior Li affinity and stability, further integration of more elaborate designs is required. For instance, the Ding’s group [120] synthesized a composite scaffold of nitrogen-doped carbon/Mg@rGO (NC/Mg@rGO) with a 3D interconnected nanofiber network structure. Specifically, Mg nanoparticles are uniformly dispersed within the network to serve as lithiophilic nucleation sites, while the rGO coating functions to stabilize the SEI. The 3D interconnected network provides ample space for Li deposition. Meanwhile, Mg nanoparticles guide preferential Li nucleation around them owing to their intrinsic Li affinity, and the rGO coating facilitates the formation of a stable interfacial layer to mitigate side reactions (Fig. 11e). As can be seen from the SEM images, significant structural differences exist among NCM, NC/Mg, and NC/Mg@rGO microspheres. The pristine NCM exhibits a relatively smooth surface, while some protrusions emerge on the surface after magnesium doping. Eventually, the RGO sheets are tightly wrapped around the NCM surface, which confirms the successful synthesis of the target structure (Fig. 11f). This composite host delivers an ultrahigh specific capacity of 1863 mAh g^−1^. The corresponding LFP//NC/Mg@rGO-Li full cells realized excellent rate and cycling performance by maintaining a high capacity of 119 mAh g^−1^ after 800 cycles at 1.0 C, validating that the composite architecture can effectively guide uniform Li deposition and suppress dendrite growth.

As evidenced by the aforementioned studies, remarkable achievements have been made in lithiophilic modification and interfacial regulation through the integration of diverse structural designs including 3D carbon-based lithiophilic scaffolds, spherical/core–shell lithiophilic structures, and 3D composite lithiophilic architectures. Compared to single structural design, multi-strategy integration effectively combines the advantages of individual strategies, yielding synergistic improvement effects. Under laboratory conditions, these designs have exhibited excellent electrochemical performance in both symmetric cells and full cells, demonstrating immense application potential of these lithiophilic carbon-based hosts in developing high-energy–density AFLMBs. However, existing schemes still fail to address the spatial directionality of Li deposition. Uneven distribution of lithiophilic sites tends to induce preferential Li deposition on the surface, leaving internal cavities underutilized. Thus, designing a rational lithiophilic gradient structure to guide directional Li deposition constitutes a critical approach to solving this problem.

### Lithiophilic Gradient Design

Above, we have discussed relatively elaborate lithiophilic structure designs, including single Cu substrate modification and carbon-based substrate modification. However, current modification strategies lack effective guidance for Li deposition. Additionally, the uniform structure of porous current collectors fails to efficiently guide Li metal deposition to the bottom, resulting in a phenomenon termed “top growing.” Different from the uniform structures discussed above, gradient design introduces spatial variations in lithiophilicity to regulate Li deposition directionally. Accordingly, this section will focus on the guiding mechanisms of various gradient lithiophilic structures on Li deposition, including conductivity gradients [139, 227], lithiophilicity gradients [186, 228], and pore size gradients [2, 229].

**Fig. 12:**
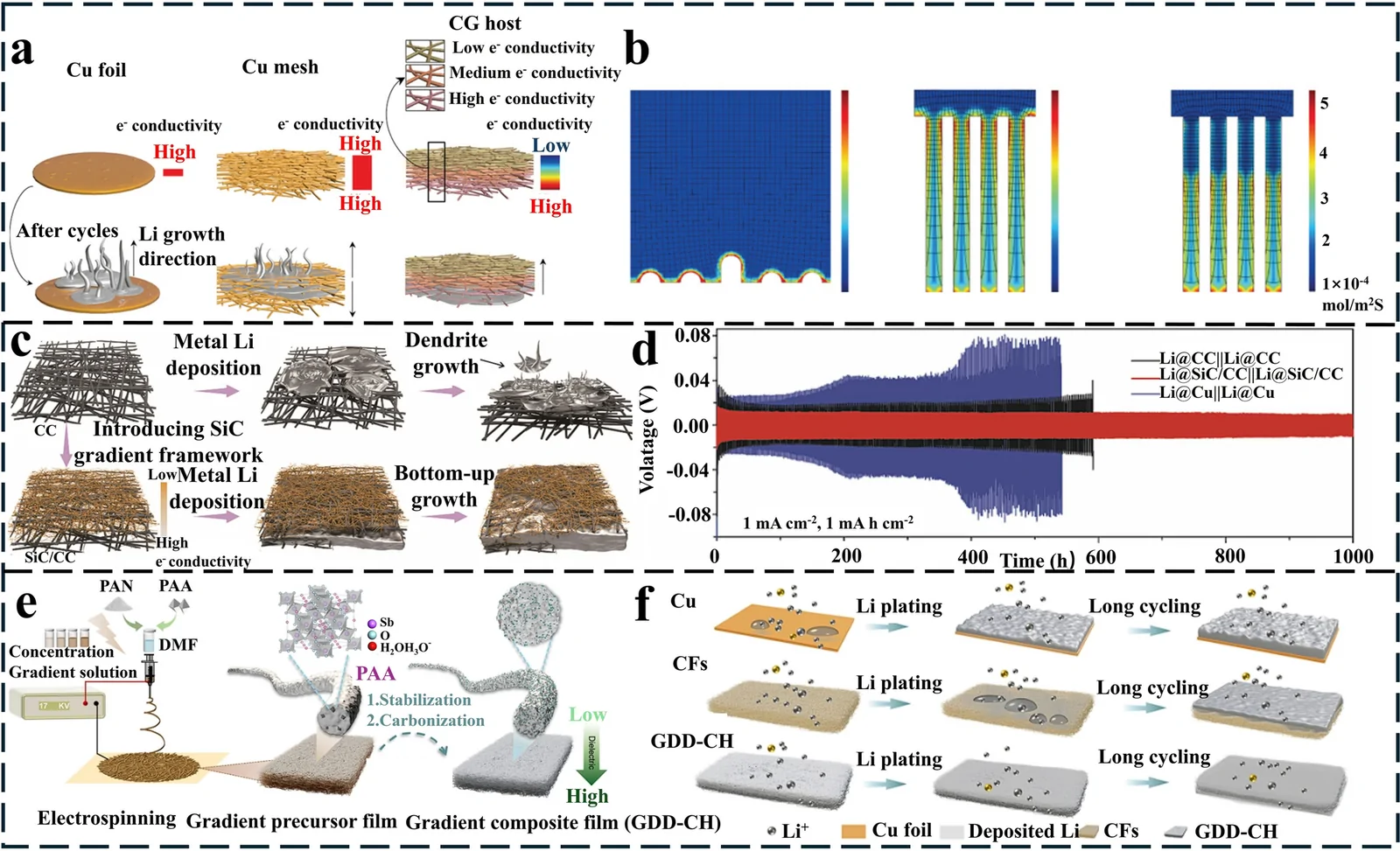
**a** Schematic diagram of the effect of Cu foil (left), Cu mesh (middle), and conductivity gradient host (right) on Li deposition. **b** Schematic diagram of Li⁺ flux via COMSOL simulation [231]. Copyright 2020 Wiley. **c** Schematic diagrams of SiC/CC and Li@SiC/CC frameworks before and after Li metal deposition. **d** Voltage–time profiles of various electrodes in symmetrical cells at a current density of 1 mA cm^–2^ for an area capacity of 1 mAh cm^–2^ [141]. Copyright 2022 Elsevier. **e** Schematic illustration of synthesis producer of GDD-CH. **f** Schematic diagrams of Li deposition on planar Cu foil (upper), CFs (middle), and GDD-CH (lower) [232]. Copyright 2024 Wiley

Conductivity gradient is to construct a gradient structure with gradually increasing conductivity from top to bottom, thereby inducing a specific potential gradient distribution in the 3D framework. Under the effect of this distribution, the bottom of the 3D framework is more prone to trap electrons compared to the top region, making it a preferred site for Li deposition and dissolution [228, 230]. Thus, constructing a conductivity gradient is recognized as an effective way to guide Li toward “bottom-up” directional deposition. However, early gradient-designed Li hosts are usually too thick to meet the requirements for high energy density or present limited performance at high current densities due to the high conductivity of the top surface. To address this, Lee et al. [231] engineered a lightweight conductivity gradient system, comprising a highly conductive bottom layer, an electrically insulating top layer, and an interlayer with moderate conductivity (Fig. 12a). Specifically, the highly conductive copper nanowires (CuNWs) serving as the bottom layer provide electron transport pathways, facilitating Li deposition, while CNFs mixed with SiO_2_ nanoparticles act as the top insulating layer to inhibit current aggregation at the top, preventing Li nucleation there. The low-conductivity interlayer (CNFs mixed with CuNWs) ensures smooth electron conductivity, enabling Li fully deposit at the bottom to further grow toward the interlayer. COMSOL multi-physics simulations confirm that the ionic reaction flux is mainly concentrated in the bottom and interlayer (Fig. 12b), validating the stability of Li plating at the bottom and suppressing Li dendrite growth induced by the tip effect.

Although this structural design displays impressive performance, single electron conductivity regulation is insufficient for Li⁺ deposition, as it assumes that Li^+^ follows the electron migration path for deposition. However, in practice, electron conductivity and ionic conductivity jointly determine Li^+^ deposition kinetics. If the conductive network fails to disperse Li^+^ deposition sites, dendrite growth will still occur. Thus, constructing a structure with electron–ion synergistic regulation is critically important. Sun et al. [141] developed a 3D topological architecture based on conductivity gradient by growing SiC whiskers on CC (denoted as SiC/CC, Fig. 12c). The SiC whiskers in the top layer act as an electronic insulation layer, eliminating the electric field effect at the top, thus preventing dendrite growth there and guiding bottom-up Li deposition. Meanwhile, its unique topological structure enhances the effective transport capability of Li^+^, enabling Li^+^ to migrate to the deepest part of the framework. Furthermore, the porous structure and strong chemical affinity of SiC/CC reduce local current density and enhance Li⁺ reaction flux, rendering Li less prone to detachment from the framework during charge–discharge cycling. As evidenced by COMSOL simulations, the architecture effectively guides Li^+^ reaction flux to the bottom, realizing bottom-up deposition. Electrochemical tests demonstrate that symmetric cells with Li@SiC/CC maintain a long cycle life of up to 1000 h and an ultra-low overpotential (20 mV) at a current density of 1 mA cm⁻^2^ (Fig. 12d), verifying excellent structural functionality.

Traditional conductive skeletons suffer from poor top-layer lithium growth and dead lithium accumulation due to internal inconsistencies caused by uneven Li⁺ concentration gradients and uniform electric field effects. Constructing a conductivity gradient based on dielectric skeletons enables more precise regulation. For instance, Zhang et al. [232] designed a 3D hierarchical architecture with gradient-distributed dielectric properties (GDD-CH), composed of Sb particles with a bottom-up decreasing gradient distribution and a 3D CF framework (Fig. 12e). This unique design effectively regulates Li⁺ deposition behavior. The bottom layer with high dielectric constantly actively attracts and homogenizes Li^+^ flux, significantly reducing the Li^+^ concentration gradient within the structure. Meanwhile, uniformly distributed Sb particles alloy with Li during cycling to form Li_3_Sb with ultrahigh ionic conductivity. This not only affords excellent lithiophilicity, reducing the Li nucleation overpotential to as low as 27 mV, much lower than that of Cu substrates (63 mV) and CF frameworks (56.5 mV), but also constructs a high-speed transport pathway for Li⁺, guiding Li to preferentially deposit inside the structure rather than on the top (Fig. 12f), markedly suppressing Li dendrite formation. The ingenious integration of dielectric gradient and lithiophilic alloy constitutes a dual-driven or multi-driven mechanism, ensuring excellent reversibility of Li plating/stripping on GDD-CH even under high deposition capacity (10 mAh cm⁻^2^) and ultra-low N/P ratio (1.51).

**Fig. 13:**
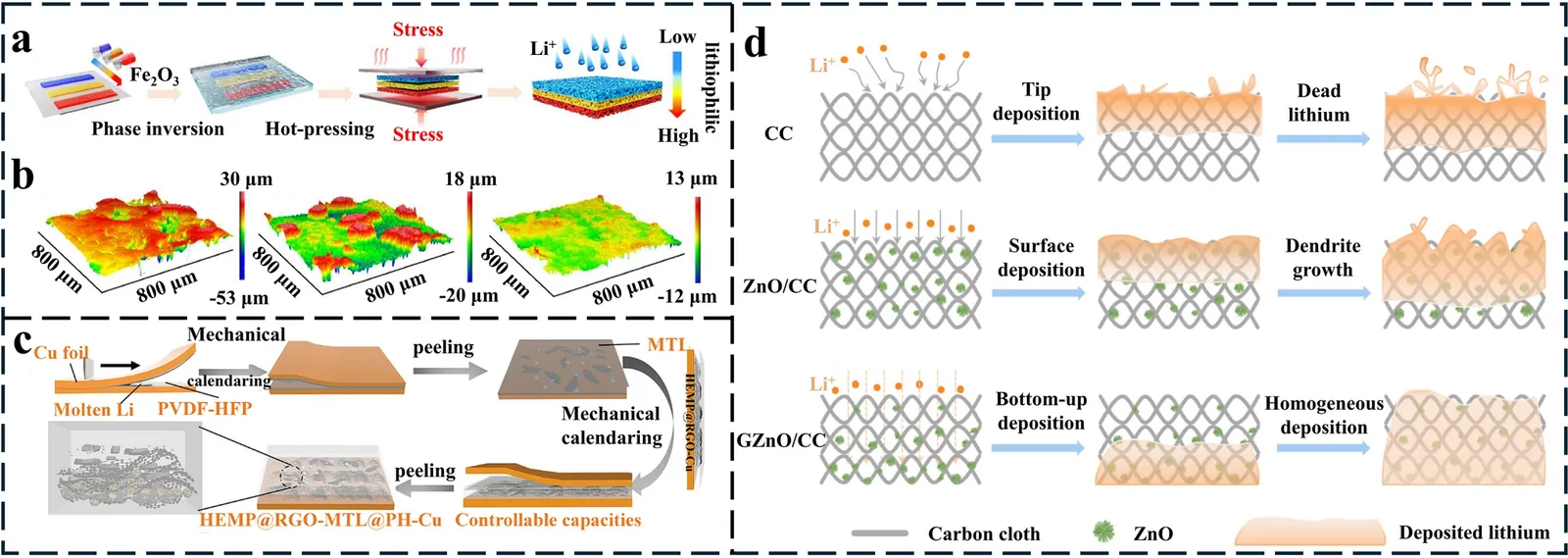
**a** Schematic diagram of the preparation process of GFC@PVDF. **b** Optical surface profile images of Cu (left), FC@PVDF (middle), and GFC@PVDF (right) [235]. Copyright 2025 Elsevier. **c** Schematic diagram of the synthesis process of the HEMP@RGO-MTL@PH-Cu composite structure [140]. Copyright 2025 Wiley. **d** Schematic diagram of the working principle of (top) CC, (middle) ZnO/CC, and (bottom) GZnO/CC in lithium deposition [138]. Copyright 2025 Elsevier

Relying solely on conductivity or dielectric gradients to guide Li⁺ deposition may be far from sufficient and may fail to precisely regulate Li nucleation sites at the more microscopic scale (e.g., atomic/molecular level [233, 234]). Thus, the introduction of lithiophilicity gradients has emerged as a critical component. Via the gradient distribution of surface energy states, it provides a bottom-up pathway for Li deposition. Lithiophilicity gradients are constructed by selecting highly lithiophilic materials at the bottom. These materials, featuring low nucleation barriers and more negative Li binding energies, facilitate effective Li⁺ adsorption to establish a direct pathway for preferential Li deposition at the bottom of the host structure. In contrast, lithiophobic materials are adopted in the top region of the host. These materials exhibit high nucleation barriers, which inhibit Li aggregation and deposition on their surfaces. This gradient design not only achieves bottom-up directional Li deposition, but also ensures Li⁺ deposition in a controllable manner, effectively mitigating dendrite growth. For conventional Cu current collectors, their lithiophobic nature gives rise to an uneven Li⁺ concentration gradient. Coupled with local electric field aggregation, this leads to Li surface deposition and uncontrolled Li dendrite growth. Li et al. [235] fabricated a 3D hierarchical flexible membrane electrode with lithiophilicity gradient (denoted as GFC@PVDF, Fig. 13a). With CNTs as the core, its excellent conductivity provides rapid electron transport. Utilizing the melting point of β-PVDF, three flexible membranes with varying Fe_2_O_3_ contents were hot-pressed together forming a bottom-up decreasing Fe_2_O_3_ concentration gradient. During cycling, Fe_2_O_3_ reacts with Li to form a hybrid electronic/ionic conductive alloy of Fe/Li_2_O, serving as a pathway for rapid charge decoupling and transport, thus enhancing rapid Li⁺ transport at the bottom. The bottom-up decreasing lithiophilicity gradient guides bottom-up Li deposition, alleviating the top-growing issue. Observation of Li deposition morphology via optical profilometry (Fig. 13b) reveals that both conventional Cu foils and non-gradated FC@PVDF electrodes exhibit a significant height difference after Li deposition, while the GFC@PVDF electrode shows a much smaller height difference, further validating the superior Li deposition guiding capability of this architecture.

For AFLMBs, the primary bottleneck lies in the lack of active Li supplementation, leading to CE decay and failure to achieve long-term cycling performance. Thus, designing an anode structure that integrates active Li supplementation, and high performance has emerged as a highly promising research direction. Ma et al. [140] synthesized a structure integrating a layer-stacked interface design with a lithiophilicity gradient. Specifically, they dispersed high-entropy metal phosphides (HEMP) into rGO via a simple wet-chemical method. Polyvinylidene fluoride-hexafluoropropylene (PVDF-HFP) was mixed with molten Li in an appropriate ratio, and a Li-supplementing top layer was formed through layer-transfer printing technology (Fig. 13c). Anchoring HEMP particles within the rGO matrix constructs a lithiophilic gradient bottom layer, which significantly reduces the nucleation overpotential. The lithiophilic elements (e.g., Sn, Sb, and P) in HEMP act as sequential “magnets” to homogenize Li^+^ influx, while the rGO scaffold alleviates mechanical stress induced by volume expansion. The pre-lithiated top layer (a 5 μm-thick MTL@PH-Cu layer) not only supplements the lost Li to boost the CE from 74.3% to 100.7%, but also regulates Li⁺ transport. Under 83% depth of discharge, symmetric cells maintain 600 h of cycling stability at a current density of 5 mA cm⁻^2^ and a high areal capacity of 10 mAh cm⁻^2^. Full cells assembled with NCM811 cathode achieve a high-capacity retention of 90.9% after 100 cycles.

Additionally, to address the risk of dendrite penetration through the separator caused by preferential Li deposition near the separator, Cui et al. [138] proposed a vertically distributed lithiophilic ZnO gradient on a 3D CC substrate (GZnO/CC) to regulate lithium deposition in a controlled “bottom-up” manner (Fig. 13d). They treated one side of CC via oxygen plasma, endowing it with single-side hydrophilicity, followed by hydrothermal synthesis of a vertically distributed ZnO lithiophilicity gradient. Specifically, ZnO reacts with Li to form Li-Zn solid solutions and Li₂O during the first charge–discharge process. The former serves as lithiophilic sites to lower nucleation energy at the bottom and homogenizes Li^+^ flux, while the latter with high ionic conductivity guides “top-down” Li deposition. Meanwhile, the 3D CC substrate disperses local current density and provides abundant Li deposition space. COMSOL simulations confirm that the gradient design suppresses the occurrence of hot-spot effects induced by Li^+^ concentration localization. Electrochemical tests further testify that symmetric cells achieve stable cycling for 2500 h, and full cells assembled with LFP cathode deliver a high-capacity retention of 90.7% after 1000 cycles. These lithiophilicity gradient structural designs have significantly enhanced the stability of Li plating/stripping and mitigated volume fluctuations during long-term cycling, providing a feasible solution for the development of high-performance AFLMBs.

**Fig. 14:**
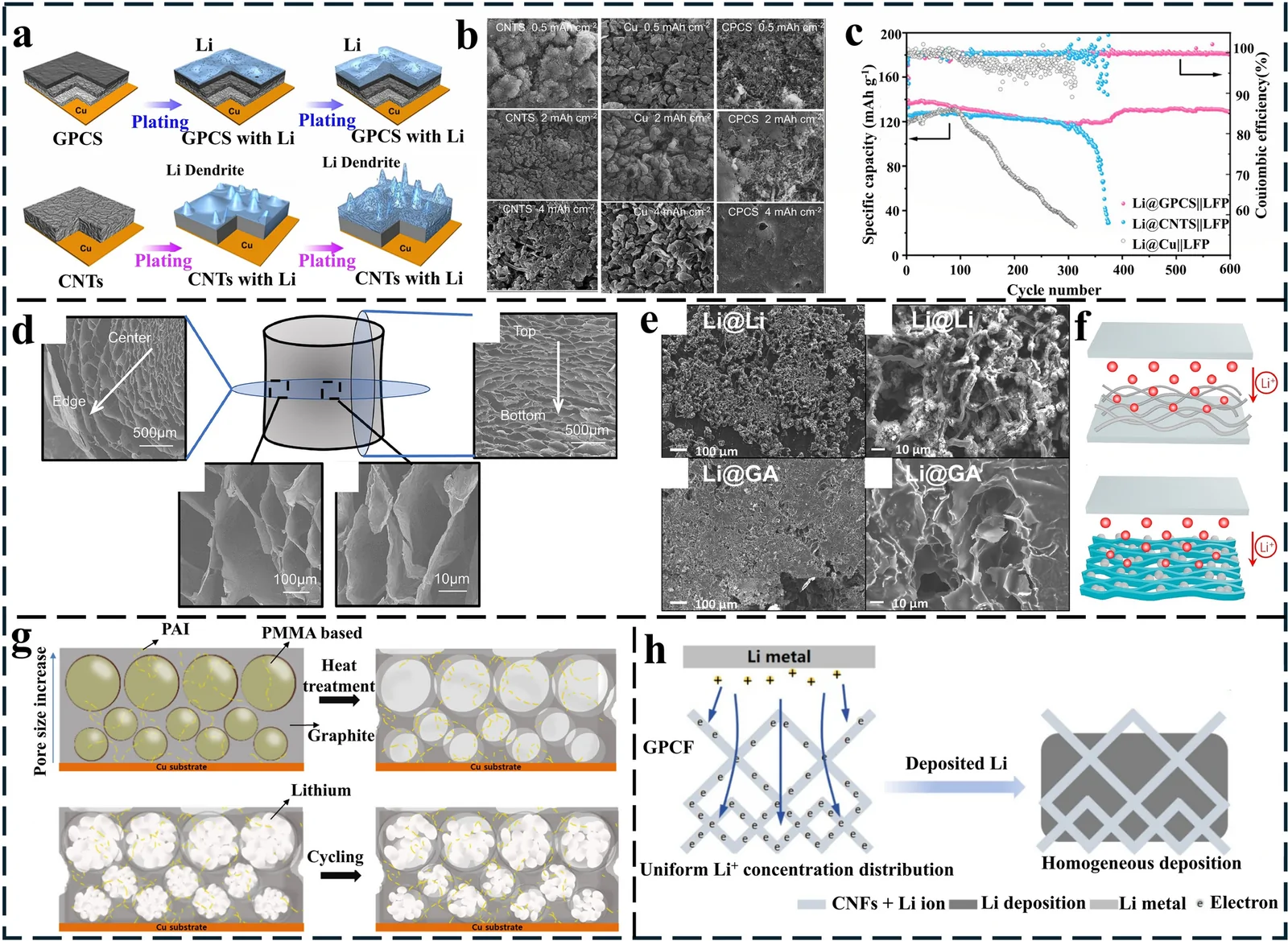
**a** Schematic diagram of Li deposition on GPCS (upper) and CNTS (lower). **b** Schematic SEM images of the electrode surface under different deposition capacities for bare Cu (left), CNTS (middle), and GPCS (right). **c** Cycling performance of the three structures after assembling full cells with LFP [238]. Copyright 2022 Wiley. **d** SEM image of the GA structure. **e** SEM images of the Li metal electrode after Li electrodeposition on the surface of (upper) Li foil and (lower) GA. **f** Schematic diagrams of Li deposition behavior modes on Li foil (upper) and GA (lower) [239]. Copyright 2022 Elsevier. **g** Schematic diagram of the three-dimensional carbon-based porous anode (3D-CPA), showing the pore size gradient (top) and the cycling behavior for fabricating the 3D-CPA (bottom) [240]. Copyright 2021 American Chemical Society. **h** Schematic diagram showing the deposition behavior of lithium metal within GPCF [237]. Copyright 2025 American Chemical Society

Different from the above conductivity and lithiophilicity gradient design, pore size gradient construction requires a depth-dependent decreasing distribution of host pore sizes. Specifically, the top region features higher porosity, while pores gradually shrink and become denser toward the bottom. In this structure, large pores in the top optimize electrolyte wettability, reducing the Li^+^ concentration gradient in the electrolyte and homogenizing Li^+^ distribution throughout the structure [236]. Meanwhile, the decreasing pore size increases the SSA per unit volume, thereby reducing the charge transfer resistance in the bottom region [237]. This favors preferential Li^+^ deposition at the bottom, followed by gradual upward filling, ultimately achieving a “bottom-up” Li deposition process. Based on this, Liu et al. [238] designed a gradient pore carbon skeletons (GPCS) via continuous casting. This was constructed by integrating three types of 1D carbon materials with different diameters: 150–180-nm CNTs for the top layer, 60–80-nm CNTs for the middle layer, and 20–30-nm CNTs for the bottom layer, leading to a unique architecture of pore size gradient decreasing with depth. Both finite element simulations and experimental analyses confirm that this structure can effectively guide Li growth and mitigate severe volume fluctuations during Li plating/stripping. Furthermore, its unique porous architecture addresses the issue of direct Li deposition on the surface of the conductive host, thereby safeguarding the functionality of the pore size gradient structure (Fig. 14a). Compared to pure CNTs, the GPCS structure enables smoother Li deposition (Fig. 14b), endowing Li-GPCS half-cells with a stable high CE of 98% over 320 cycles at 1 mAh cm⁻^2^. In addition, to further investigate the electrochemical performance, Li@GPCS, Li@CNTS, and Li@Cu anodes were paired with LFP cathodes and assembled into full cells. Among these, the cycling stability of the Li@GPCS full cell is far superior to that of the Li@CNTS and Li@Cu counterparts; the CE and specific capacity of the cell equipped with Li@GPCS can be maintained at 99.9% and 130 mAh g⁻^1^ after 600 cycles (Fig. 14c).

Inspired by these works, the pore size gradient design has been further extended to high-performance LMBs and AFLMBs. Quan et al. [239] engineered a graphene aerogel (GA) with 3D porous structure as a high-performance LMA host to achieve dendrite-free and uniform Li deposition. The GA was fabricated via self-assembly of rGO, natural drying, and subsequent annealing, featuring a continuous pore size gradient from the center to the edge of the material (Fig. 14d). This structure provides efficient ionic/electronic transport pathways and introduces pyridinic N and pyrrolic N functional groups via annealing as well. These groups act as lithiophilic sites to reduce Li nucleation barrier. It can be clearly observed from the SEM images that Li deposition on the GA anode is significantly more compact and uniform compared with that on the bare Li anode (Fig. 14e). Additionally, the small pores at the bottom afford an ultra-high SSA, dispersing local current density and suppressing dendrite growth. In contrast, the large pores near the top enhance Li^+^ transport kinetics. These observations indicate that, compared with bare metallic Li, the 3D GA scaffold with a unique gradient hierarchical porous structure can effectively homogenize the distribution of Li⁺ ions and suppress the growth of lithium dendrites (Fig. 14f). Furthermore, the morphology and deposition amount of Li were precisely controlled via electrochemical deposition, achieving a high Li/C mass ratio of up to 5.0. Benefiting from these advantages, the Li-GA electrode delivers an ultra-high deposition capacity of 2500 mAh cm⁻^2^ in symmetric cell tests, fully demonstrating its great practical potential. Building on this, Choi’s group [240] achieved practicalization and scaling of pore size gradient structures via a commercial slurry process using PMMA as the pore-forming template, fabricating a 3D carbon-based porous anode (3D-CPA) with a bottom-up gradient. The top large pores enhance electrolyte wettability and Li⁺ transport, while the bottom small pores provide abundant Li deposition sites to suppress dendrite growth (Fig. 14g). Additionally, the well-defined pores mitigate volume expansion/contraction during Li plating/stripping and ensure unobstructed electronic transport.

The adoption of a vertical gradient pore size distribution structure with small pores at the bottom and large pores at the top can effectively inhibit the top growth of lithium dendrites. Early studies based on this idea used graphite to construct the pore size framework, and subsequent researchers further innovated by selecting carbon nanofibers as the base material for the pore size structure. Specifically, Zhang et al. [237] designed a conductive carbon nanofiber carrier (named GPCF) with an oriented gradient pore structure, which consists of two layers of carbon nanofiber networks: One layer is a small pore size structure (approximately 2.6 μm) used as the current collector side; the other layer is a large pore size structure (approximately 3.9 μm) facing the separator side. Benefiting from this unique structure, a uniform lithium-ion flow with a gentle ion concentration gradient from the separator side to the current collector side is formed inside the GPCF, thereby achieving a dendrite-free lithium deposition morphology (Fig. 14h). Electrochemical test results show that the GPCF@Li||LFP full cell with GPCF as the anode can still maintain a high-capacity retention rate of more than 70% after 370 cycles at a charge–discharge rate of 0.5 C, and at the same time, the CE is stably maintained at a high level of 99.5%.

In summary, lithiophilic gradient design enables bottom-up directional lithium deposition by precisely tailoring the gradient variations in conductivity, lithiophilicity, and pore size distribution within 3D frameworks. This strategy not only effectively suppresses lithium dendrite growth and reduces inactive lithium accumulation but also mitigates volume fluctuations during prolonged cycling. However, from an engineering perspective, the manufacturing complexity and scalability of such designs remain significant challenges. Constructing gradient architecture typically relies on precision techniques such as multilayer coating, template-assisted methods, or vapor deposition, which not only increase fabrication complexity but also impose higher demands on the continuity and consistency of roll-to-roll production. Compared to conventional homogeneous 3D frameworks fabricated via one-step or mixing methods, gradient designs currently lack clear advantages in simplifying manufacturing processes and reducing costs. Nevertheless, in terms of performance, gradient designs achieve effects that traditional homogeneous frameworks can hardly match. While conventional 3D structures offer a large SSA to reduce local current density, they struggle to fundamentally resolve issues such as surface pore sealing and low internal pore utilization caused by “top-down” lithium filling. By spatially modulating lithiophilicity and conductivity, gradient designs induce preferential lithium deposition at the bottom of the framework, enabling efficient utilization of lithium resources and buffering volume changes a mechanism that demonstrates superior stability under long-term cycling and high areal capacity conditions. Unfortunately, they may compromise overall energy density due to the introduction of thick buffer layers or heavy lithiophilic precursors. This trade-off must be carefully evaluated against the performance of conventional hosts. Taken together, the core value of lithiophilic gradient design lies in its active regulation of lithium deposition behavior, offering enhanced failure suppression compared to traditional passive physical confinement strategies. However, to achieve large-scale application, breakthroughs are needed in simplifying fabrication processes, reducing costs, and improving process compatibility. Therefore, continuous exploration of material systems that offer significant gradient benefits through simple and scalable processes represents a key direction for developing high-performance gradient materials compatible with AFLMBs.

### Synergistic Design of Lithiophilic Carbon-Based Hosts

**Fig. 15:**
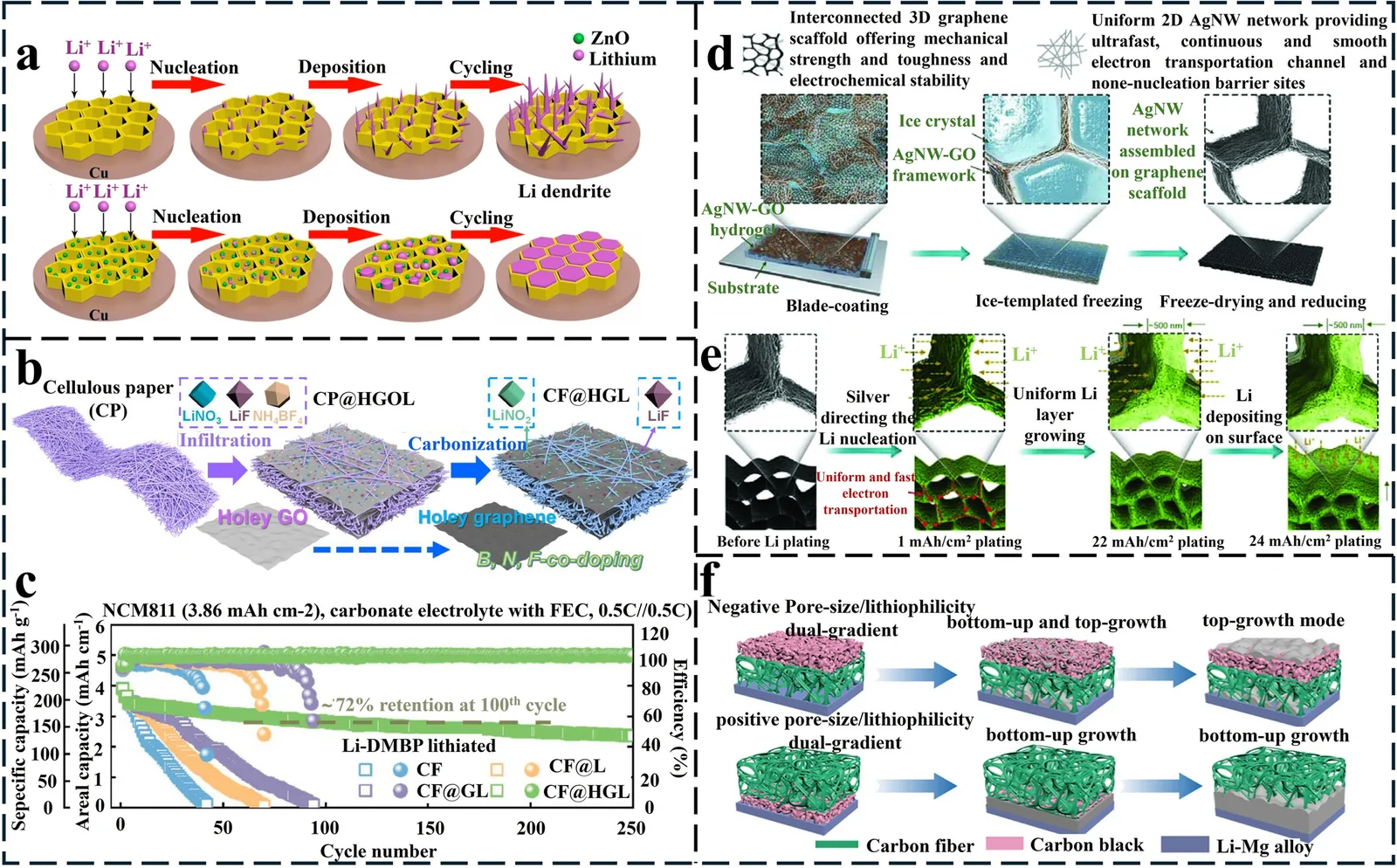
**a** Schematic diagram of the effect of the ZnO@HPC electrode structure on Li deposition [242]. Copyright 2017 Elsevier. **b** Schematic illustration of the preparation processes of CF@HGL film. **c** Charge/discharge cycling performances of CF@HGL||NCM811 full cells [243]. Copyright 2025 Elsevier. **d** Schematic diagram of the preparation process of 3D-AGBN via ice-templating method. **e** Schematic diagrams of Li deposition in 3D-AGBN under different states [244]. Copyright 2018 Wiley. **f** Schematic illustration of lithium deposition behavior in the negative pore size gradient (top) and positive pore size gradient (bottom) [236]. Copyright 2025 American Chemical Society

Building on the previously discussed lithiophilic gradient designs, further advancements have been achieved through the combination between gradient designs and lithiophilic structure engineering, framework design, and surface decorating. These strategies provide effective approaches for mitigating volume effects and stabilizing the SEI from the perspectives of 3D scaffold construction and interfacial chemical modification, respectively [105, 241]. However, under practical operating conditions, AFLMBs still face the interplay of dead Li accumulation [142], uneven nucleation [210], and poor interfacial stability [66]. Single modification strategies are no longer sufficient to achieve the synergistic optimization of both uniform deposition and long cycle life. By complementing various lithiophilic designs, a multifunctional hybrid interface integrating lithiophilic chemical guidance, uniform electric field distribution, and mechanical buffering capability is constructed. This design enables multidimensional regulation of lithium deposition behavior in AFLMBs from an interfacial synergy perspective. Jin et al. [242] designed a dendrite-free LMA using a bamboo-derived 3D hierarchical porous carbon scaffold decorated with ZnO quantum dots (Fig. 15a). The porous structure reduces local current density and buffers volume expansion, while the ZnO quantum dots serve as lithiophilic sites that react with Li to form Li₂O and Zn, subsequently generating Li-Zn alloys. This guides Li to preferentially nucleate and grow within the pores, thereby preventing disordered surface deposition.

Although the ZnO modification improves deposition uniformity, the coating suffers from structural instability during long-term cycling. Guo’s group [243] designed a hierarchical carbon framework (CF@HGL) composed of cellulose-derived carbon fibers, heteroatom (B, N, F)-doped holey graphene and pre-lithiated lithium salts (LiF and LiNO₃) as the host electrode for AFLMBs (Fig. 15b). In this design, the holey graphene provides abundant nucleation sites and lowers the Fermi level, while heteroatom doping induces the formation of a stable SEI rich in LiF/Li₃N. Meanwhile, chemical pre-lithiation increases the initial CE to over 100%. This design enables anode-free full cells paired with an NCM811 cathode to achieve a capacity retention of 72% after 100 cycles at 1.93 mA cm⁻^2^ (Fig. 15c), with an average CE of approximately 99.8%, showing certain potential of practical application. In another work, Xue et al. [244] designed a hierarchical porous host (3D-AGBN) composed of silver nanowires (AgNWs) integrated with a 3D graphene skeleton (Fig. 15d). The fabrication process began with blade-coating a viscous ink containing AgNWs and GO nanosheets to form an AgNW-GO hydrogel film, followed by ice-templated freeze-drying to generate a binary network structure. The self-assembly of AgNWs into a continuous macroscopic two-dimensional conductive network significantly facilitates electron transport. Leveraging the low nucleation overpotential of Ag and the advantages of the self-assembled highly conductive 2D network, metallic Li can be directly deposited within the scaffold, achieving uniform deposition (Fig. 15e). Meanwhile, the interconnected graphene skeleton endows the electrode with excellent mechanical strength and toughness to buffer stress fluctuations during cycling. Benefiting from this synergistic design, symmetric cells using the Li@3D-AGBN anode achieve stable cycling for over 1000 cycles at an ultrahigh current density of 40 mA cm⁻^2^, with an overpotential below 120 mV. Nevertheless, the preparation process involves multiple complex steps, including AgNW synthesis, ice-templated freeze-drying, and hydrazine hydrate reduction, which entail high costs and significant challenges for large-scale production, making its industrial application a substantial challenge.

In addition, the combination of gradient design and lithiophilic site modification represents another effective strategy to inhibit the top growth of lithium dendrites. Song et al. [236] proposed a dual-gradient design by infiltrating molten Li-Mg alloy into a double-layered carbon paper (DLCP) substrate consisting of a macroporous CF layer and a microporous carbon black (CB) layer (Fig. 15f). In this design, the DLCP provides a pore size gradient, while the Li-Mg alloy introduces a lithiophilic gradient. The macroporous CF layer ensures mechanical integrity, and the microporous CB layer reduces local current density to suppress dendrites. Notably, the Li-Mg alloy exhibits excellent lithiophilicity, which lowers the Li nucleation barrier. The pore size gradient, combined with the lithiophilicity of the Li-Mg alloy, promotes a “bottom-up” Li plating mode. Although this design demonstrates good performance, several defects cannot be ignored: The Li-Mg alloy provides lithiophilicity, but Mg may undergo dissolution, migration, or structural reorganization during repeated Li deposition/stripping, potentially compromising or invalidating its lithiophilicity. Additionally, the interfacial bonding strength among the bilayer structure (CF and CB layers) and the Li-Mg alloy requires further investigation, as interfacial delamination during long-term cycling may lead to performance degradation.

In summary, the synergistic design of lithiophilic carbon-based hosts exhibits multidimensional regulation potential, yet it is also constrained by several critical limitations: 1) Excessive modification with lithiophilic sites may disrupt the original structure of the carbon-based skeleton, leading to reduced conductivity and mechanical collapse of the 3D framework; 2) complex hierarchical structures often rely on overly intricate synthesis routes, which not only increases the difficulty and cost of fabrication, but also introduces uncontrollable interfacial side reactions. Therefore, striking a balance between functional synergy and structural simplification and avoiding over-design are crucial for the practical application of interfacial synergistic design.

### Operando Characterization for AFLMBs

To date, advanced characterization techniques have become increasingly important. Among these, operando characterization is crucial for real-time monitoring of lithium deposition behavior and morphology, lithium dendrite growth, SEI derivation, and evolution and structural changes in AFLMBs based on carbon hosts. To fundamentally address the challenges associated with carbon-based hosts in AFLMBs, the employment of operando characterizations is essential to monitor the dynamic electrochemical processes. Integrating these advanced characterization techniques offers a comprehensive pathway to optimize the design of carbon-based hosts for AFLMBs.

**Fig. 16:**
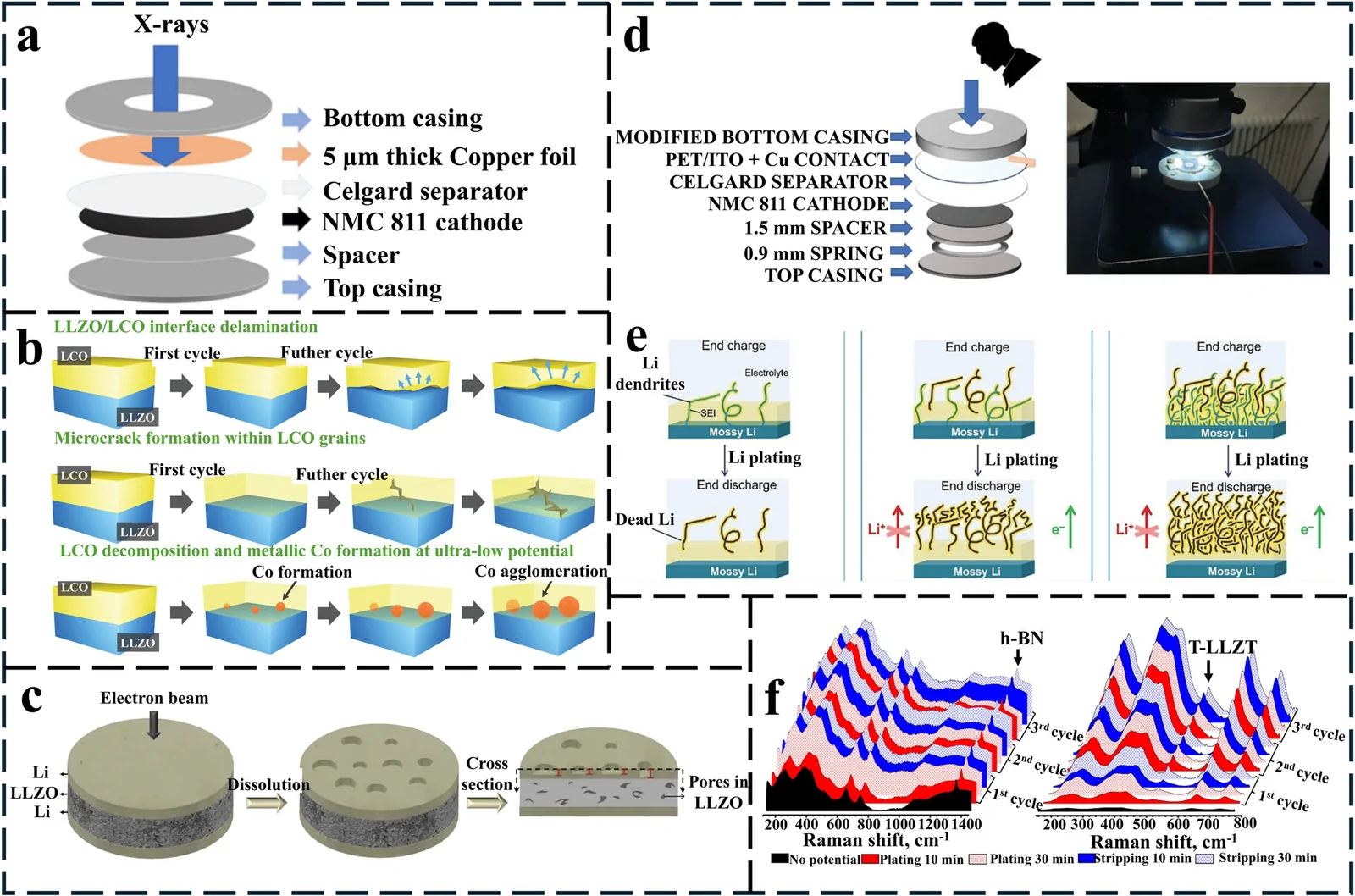
**a** Schematic diagram of in situ XRD cell [245]. Copyright 2022 Elsevier*.* **b** Schematic of the Solid-state lithium batteries capacity degradation mechanisms during electrochemical cycling [247]. Copyright 2022 Wiley. **c** Schematic of cell behavior after the dissolution [248]. Copyright 2022 Springer Nature.** d** Schematics and images of the operando cycling setup. **e** Schematics representing the mechanism of Li dendrite nucleation (left), growth (middle), and dead Li formation (right) in the first, second, and third discharge cycles, respectively [250]. Copyright 2024 Wiley. **f** In situ Raman analysis during galvostatic plating/stripping of (left) h-BN-coated and (right) uncoated LLZT [252]. Copyright 2020 American Chemical Society

Operando XRD serves as a powerful tool to track the phase evolution and crystallinity of lithium during plating and stripping. Zhu et al. [245] employed in situ XRD to observe the accumulation of dead lithium along the (110) crystallographic plane on a 5-μm-thick Cu foil in anode-free Cu/NCM811 batteries (Fig. 16a). The results revealed that during charging, Li⁺ was extracted from the cathode and deposited onto the Cu foil, leading to an enhanced Li (110) peak. Upon discharging, Li was oxidized to Li⁺ and re-intercalated into the cathode, resulting in a weakened Li (110) peak. Concurrently, the amount of dead lithium increased with cycling frequency. These findings demonstrate that dead lithium formation is a critical factor contributing to rapid CE decay and battery failure. A key advantage of this method lies in its non-destructive nature, enabling continuous data acquisition during battery cycling and providing authentic insights into the dynamic phase evolution of the electrodes. Notably, high-energy XRD has also been employed to identify the formation of Li_x_C_y_ intermediate phases in graphite anodes under extreme fast charging conditions [246]. This provides valuable insights into understanding the early-stage lithium nucleation mechanism and offers critical guidance for the design and optimization of AFLMBs. Hou et al. [247] employed in situ transmission electron microscopy (in situ TEM) to observe, in real time, the deintercalation, and intercalation processes of lithium in the cathode material (LiCoO₂, LCO) within all-solid-state garnet-type electrolyte (Li₇La₃Zr₂O₁₂, LLZO) lithium batteries. Their observations revealed that the deintercalation and intercalation of Li⁺ induce changes in the lattice parameters of the cathode material. Under over-discharge conditions, excessive Li intercalation into LCO leads to structural collapse at the electrode interface (Fig. 16b). Furthermore, during cycling, the volume changes of LCO resulting from Li deintercalation/intercalation generate periodic compressive/tensile stresses at the LLZO/LCO interface, causing debonding an interfacial degradation behavior analogous to the mechanical failure mechanisms observed in SEI layers.

For a deeper understanding of the nucleation mechanism, operando TEM allows for real-time observation of atomic-scale structural changes. The in situ TEM observations can directly capture the nucleation sites, growth pathways, and ultimate morphologies of Li on these substrates. Additionally, this technique enables investigation of interfacial interactions, such as the preferential nucleation of Li at defect sites on carbon-based hosts. However, the electron beam damage inherent to in situ TEM cannot be overlooked. As noted by Hou et al., under high-vacuum conditions, electron beam irradiation may induce localized sample heating, radiation damage, or unintended non-electrochemical reactions, all of which increase the complexity and difficulty of interpreting in situ observations. Golozar et al. [248] adopted in situ SEM to monitor the interfacial behavior between lithium metal and LLZO electrolyte during pressurized cycling. They observed that lithium dissolution did not occur uniformly but preferentially took place at grain boundaries and defect sites, leading to localized thinning of the lithium layer (Fig. 16c). Subsequently, protrusions and two distinct dendrite morphologies mossy and needle-like were clearly observed near the thinned regions. Compositional analysis revealed the presence of Li, C, and O elements within the dendrites, suggesting a possible correlation with compounds such as Li_2_CO_3_, Li_x_C_y_, and Li_2_O. Furthermore, in another work by the authors [249], focused ion beam–scanning electron microscopy (FIB-SEM) combined with EDS was employed to conduct cross-sectional analysis of the “island” regions formed on the lithium anode surface after cycling. Elemental mapping revealed the enrichment of sulfur, fluorine, and nitrogen around these islands, indicating the decomposition of LiTFSI salt and the formation of compounds such as Li_3_N, LiF, and Li_x_SO_y_. This finding reveals that the decomposition of the salt at grain boundaries and defect sites serves as a crucial factor inducing local lithium consumption and lithium dendrite nucleation.

Morphological evolution is critical for evaluating host effectiveness. Operando SEM and optical microscopy have been widely utilized to visualize dendrite growth and the volume change of the anode interface during cycling. Notably, a key advantage of in situ SEM lies in its ability to directly observe morphological changes on the lithium electrode surface with submicron-scale resolution. When combined with EDS, it enables simultaneous monitoring of elemental distribution, thereby allowing for integrated analysis of morphology and chemical composition. Romio et al. [250] designed a customized CR2032 coin cell featuring a transparent window made of indium tin oxide-coated polyethylene terephthalate (ITO-PET) to enable macroscopic visualization of lithium deposition under various C-rates via in situ optical microscopy (Fig. 16d). Their study revealed that at a low C-rate (C/40), lithium preferentially nucleated at defect sites, with deposition exhibiting localized enrichment and overall non-uniformity. In contrast, at a high C-rate (2 C), rapid electrolyte decomposition led to the formation of a dense SEI layer and lithium nucleated uniformly in granular form. However, insufficient ion diffusion readily induced hollow structures within the deposited layer, resulting in a seemingly uniform surface but a porous interior. Furthermore, dead lithium accumulated layer by layer during cycling, indicating poor reversibility (Fig. 16e). Based on these findings, we propose that in situ optical microscopy can serve as a rapid evaluation tool for assessing the effectiveness of carbon-based hosts in regulating lithium deposition behavior in AFLMBs. By enabling real-time observation of the spatial distribution of lithium deposition, it allows determination within very few cycles of whether a carbon-based host effectively suppresses localized aggregation. When combined with computer vision analysis, the dispersion of deposited lithium can be rapidly quantified, and a sufficiently low dispersion value would indicate an efficient host.

Additionally, operando Raman spectroscopy provides valuable insights into the structural integrity of the carbon matrix and the chemical composition of the SEI layer. In situ Raman spectroscopy enables semi-quantitative analysis of the generation rate [251]. Based on the findings of Rajendran et al. [252], LLZO rapidly forms a Li_2_CO_3_ layer upon exposure to air. However, due to insufficient quantitative analysis of its formation rate, the semi-quantitative analysis of the generation rate provided by in situ Raman spectroscopy emerges as an effective approach. Furthermore, in situ Raman analysis revealed that after introducing a hydrophobic boron nitride (h-BN) interlayer at the LLZO/Li interface, no structural or chemical changes were detected, whereas unmodified LLZO underwent a cubic-to-tetragonal phase transition (Fig. 16f). From this, it can be inferred that in situ Raman spectroscopy provides direct experimental evidence for real-time monitoring of SEI formation, phase transitions and by-product generation. Therefore, we propose that in the context of carbon-based hosts, in situ Raman spectroscopy can be employed to track peak shifts of carbon-based materials, thereby indirectly inferring Li⁺ deintercalation/intercalation behavior and the formation of Li_x_C_y_ phases.

## Practical Considerations

The above-discussed strategies addressing the design of 3D lithiophilic carbon-based hosts for AFLMBs have demonstrated superiority in performance improvement. However, the key issues faced in its practical application still require further explorations. The following sections will discuss key aspects associated with practical AFLMBs and propose potential solutions to address these challenges.

### Scalable Preparation Cost and Process Limitation

Currently, significant differences exist in the scalability of fabrication processes for high-performance 3D lithiophilic carbon-based hosts. 1) CVD technology can produce high-quality multilayer graphene as discussed earlier, but its reliance on high-vacuum conditions limits its suitability for continuous production; 2) electrospinning enables the large-scale preparation of CFs, yet it faces considerable challenges in achieving large-area uniformity and solvent recovery; 3) template methods offer substantial advantages in precisely controlling pore structures. However, the template removal process is time-consuming, labor-intensive, and generates waste liquid, which inevitably increases the complexity of continuous production. Consequently, developing roll-to-roll continuous production processes represents a promising approach to addressing challenges related to substrate transport and uniform doping [253]. Beyond scalability, the costs associated with different carbon-based materials also vary considerably. For instance, high-purity graphene commonly used in laboratories is prohibitively expensive, far exceeding the cost of low-cost raw materials such as commercial CC or biomass-derived carbon. Moreover, synthetically produced materials like AgNPs entail high raw material costs and low synthesis efficiency. Compared with commercially available LIBs, the overall cost of these carbon hosts remains substantially higher. Thus, reconciling lithiophilicity with cost reduction to narrow the gap with LIBs presents a formidable challenge for the commercialization of AFLMBs. To overcome these bottlenecks, it is essential to prioritize low-cost raw materials, such as commercial CC and biomass-derived carbons (e.g., bamboo, cotton, etc.), and to reduce fabrication costs through one-step doping or in situ composite modification strategies (e.g., loading lithiophilic metal particles via solution soaking, etc.). Simultaneously, industrially scalable production techniques, including 3D printing and template methods, should be advanced to streamline processes and enhance material consistency.

### Lithiophilicity Degradation

During long-term cycling, lithiophilic sites (e.g., heteroatom-doped sites, metal nanoparticle attachments, etc.) may be consumed or covered due to repeated rupture and reconstruction of the SEI layer, resulting in gradual attenuation of lithiophilic activity and ultimately inducing uneven Li deposit and dendrite regrowth. Additionally, pores in the 3D framework may be blocked by dead Li accumulation, reducing the effective SSA and exacerbating volume expansion and cycle performance deterioration. It is therefore necessary to develop lithiophilic sites with self-healing capability to suppress lithiophilicity degradation during long-term cycling. Specifically, interface regulation (e.g., constructing artificial SEI layers, introducing electrolyte additives, etc.) can further enhance the interfacial compatibility between the host and the SEI layer, reducing the consumption of lithiophilic sites by side reactions. Meanwhile, optimizing the pore structure of the 3D framework (e.g., hierarchical pore size design, etc.) can mitigate dead Li accumulation and maintain a stable effective SSA over long cycles. In a word, it is necessary to integrate multiple approaches to maintain the long-term effectiveness of lithiumphilic sites.

### Interface Compatibility

Poor interface compatibility between lithiophilic carbon-based hosts and electrolytes tends to induce unstable SEI layer, with more pronounced side reactions under harsh conditions such as high current density and wide temperature ranges. Besides, reaction products between some lithiophilic modified materials and Li may degrade interfacial ionic transport kinetics, leading to increased interfacial impedance. In anode-free systems, existing hosts suffer from uneven Li distribution during high-capacity deposition. Meanwhile, the weak interfacial bonding force with bare Cu current collectors easily causes increased contact resistance during cycling. Regarding this, targeted optimization strategies are required, including developing more precise lithiophilic microstructures to improve Li utilization and deposition uniformity, synergizing with high-lithium-reserve cathodes for structural matching design to accurately balance Li storage capacity and transport kinetics between the cathode and anode, as well as expanding compatibility research with solid electrolytes, leveraging the high electrical conductivity of lithiophilic carbon-based hosts to compensate for the insufficient ionic conductivity of solid electrolytes.

### Electrolyte Compatibility

#### Liquid Electrolyte

Electrolytes play a critical role in lithium nucleation and growth. The main issue with LMA is the lack of a suitable electrolyte for its development. New electrolytes need to be developed that are derived from abundant elements, manufactured at low cost and environmentally sustainable. In liquid electrolytes, based on the choice of solvent, they can be categorized into: 1) carbonate-based solvents, such as EC, DEC, propylene carbonate and dimethyl carbonate (DMC); 2) ether-based electrolytes, including dimethoxyethane (DME) and 1,3-dioxolane (DOL). Due to their distinct physicochemical properties, these two types of electrolytes exhibit diverse application scopes [44, 254]. Among them, carbonate-based electrolytes are widely used in commercial LIBs due to their wide electrochemical stability window and low cost [255]. In contrast, ether-based electrolytes are employed in batteries compatible with LMA, such as Li–S and Li-O_2_ batteries, owing to their favorable interfacial stability with lithium metal, thereby enabling reversible redox reactions in these systems [256]. In addition, novel electrolytes currently under investigation include localized high-concentration electrolytes (LHCE) and weakly solvating electrolytes (WSE). LHCE can reduce electrolyte viscosity and improve wettability [257], with attributes such as high oxidation resistance, stable interface formation, low flammability, a wide operating temperature range and low viscosity. LHCE has been widely applied in alkali metal batteries, including LMBs [258, 259]. WSE, on the other hand, provides a unique platform for investigating fundamental processes such as cation de-solvation, ion transport and interphase formation [260–262]. Their distinctive solvation environment alters the thermodynamics and kinetics of interfacial reactions, enabling the application of such electrolytes under demanding operating conditions, including high voltage or low temperature [263, 264]. Despite their widespread use in batteries, liquid electrolytes face growing limitations in terms of safety, performance and environmental impact [265–268], and their inherent flammability, risks of thermal runaway and dendrite growth as well [269], all of which compromise battery performance. Furthermore, the extraction of electrode materials compatible with liquid electrolytes such as Li and Co significantly increases the cost of liquid electrolyte-based batteries. The end-of-life disposal and recycling of these batteries further exacerbate environmental concerns [270, 271].

#### Solid-state Electrolyte

Existing solid-state electrolyte systems can be classified into crystalline and glassy states based on their crystallinity. Various crystalline electrolytes include LISICON, NASICON, perovskite and garnet-type materials [272, 273]. Glassy electrolytes include sulfide-based, oxide-based, nitride-based and LiPON-type materials, among others [274–278]. While offering improved safety to a certain extent, they generally suffer from incompatibility issues at the interface with LMA. These challenges manifest as severe interfacial side reactions, lithium dendrite penetration along grain boundaries, and electrolyte decomposition and failure during cycling [279, 280]. Among these, sulfide electrolytes exhibit a high room-temperature ionic conductivity of up to 10⁻^2^ S cm⁻^1^. However, they suffer from poor reductive stability against LMA, readily forming a mixed conductive interface [277, 281]. In contrast, oxide-based electrolytes possess high chemical stability but are characterized by brittleness, poor interfacial contact and exacerbated reactions with LMA at elevated temperatures, which can lead to structural collapse of the electrolyte. For instance, Paolella et al. [282] confirmed the reduction of Ge^4^⁺ to Ge⁰ in LAGP at 80 °C. The root cause of these issues lies in the fact that the design of existing electrolyte materials often pursues single performance metrics while neglecting synergistic compatibility with the LMA. Therefore, next-generation electrolytes tailored for AFLMBs must maintain low cost and environmental-friendliness. Electrolyte components should be selected from earth-abundant and low-cost elements, ideally derived from elements enriched in the Earth's crust, to reduce reliance on rare metals (such as Ge, La, Y, etc.) and lower raw material costs. Furthermore, synthesis processes should be simplified and scalable, avoiding high-energy-consumption and high-pollution post-treatment procedures to achieve cost-effective manufacturing. Lastly, the entire lifespan of the electrolyte must ensure environmental sustainability, encompassing non-toxic and harmless components, potential for recyclability, and minimal ecological impact during production and use. Notably, emerging systems such as halide electrolytes (e.g., Li₃InCl₆) [283, 284] and anti-perovskite structures (e.g., Li₃OCl) [285, 286] have demonstrated preliminary potential. These materials not only utilize cost-effective elements such as Al, Cl, and O, but also offer wide electrochemical windows and favorable interfacial compatibility.

### Transition from LMBs to AFLMBs

While LMA has been the standard anode for high-energy–density batteries, its application is limited by high manufacturing costs and safety concerns. Consequently, the field is gradually shifting toward AFLMBs, which eliminate the need for excess lithium during cell assembly [44, 45]. This transition not only maximizes energy density but also aligns with the goals of sustainable development [3]. Recent studies have further demonstrated innovative strategies to overcome the initial lithium deficiency in AFLMBs, paving the way for their practical viability [287]. With their unique structural design and performance advantages, AFLMBs have emerged as a primary development direction for next-generation LMBs. Conventional LMBs require LMA as the anode, with the scarcity of lithium resources and the associated purification and processing contributing to persistently high costs. Additionally, the transportation, storage, and surface treatment of lithium foil entail further expenses. In contrast, AFLMBs do not utilize a LMA. They are consisting solely of a lithium-containing cathode, electrolyte, separator, and current collector, thereby eliminating the procurement and processing costs associated with lithium foil. Furthermore, the absence of lithium foil in AFLMBs significantly simplifies the recycling process. This characteristic endows AFLMBs with a pronounced cost advantage over their entire lifespan, aligning well with the cost-effectiveness requirements for large-scale applications [45].

One effective approach to enhancing the safety of conventional LMBs is to reduce the amount of lithium metal used. Due to its high chemical reactivity, lithium metal readily induces dendrite growth and parasitic reactions with the electrolyte during cycling, leading to safety hazards such as short circuits and thermal runaway. In contrast, AFLMBs contain no active lithium in their initial state. Lithium ions are only extracted from the lithium-containing cathode and deposited onto the anode current collector during the first charge. This significantly reduces the exposure of lithium metal to the electrolyte, substantially mitigating the aforementioned safety risks [3]. Excess lithium is commonly employed in conventional LMBs to compensate for irreversible Li⁺ loss at the cathode. However, the inefficient utilization of lithium inventory ultimately reduces the practically achievable gravimetric/volumetric energy density of LMBs. Moreover, the pre-inserted lithium foil in LMBs adds inactive mass and volume to the cell, and due to the inherently low utilization efficiency of lithium metal, the actual energy density struggles to approach its theoretical limit. In contrast, AFLMBs directly eliminate the LMA, substantially reducing both the mass and volume of the battery. During charging, Li⁺ is deposited directly onto the anode current collector, minimizing the space occupied by inert components and enabling a significantly higher energy density compared to conventional LMBs [44].

The fabrication of conventional LMBs necessitates stringent control over the thickness and uniformity of the lithium foil. Moreover, lithium foil is prone to oxidation, requiring processing to be conducted entirely under an inert atmosphere, which substantially increases process complexity. In contrast, AFLMBs eliminate the entire suite of lithium foil-related procedures, thereby simplifying the assembly process. Furthermore, the study by Tron et al. [287] moved beyond conventional current collector choices, demonstrating that stainless steel, nickel, aluminum, and Al/C exhibit good electrochemical compatibility with sulfide solid-state electrolytes. This finding indicates that in AFLMBs, the current collector need not be directly compatible with LMA. It also provides evidence that AFLMBs allow the use of more chemically inert and lower-cost current collectors (e.g., stainless steel) for direct assembly with solid electrolytes, thereby simplifying the anode-side fabrication process and substantially streamlining battery manufacturing at its source.

### Technology Roadmap and Commercialization Prospects

While numerous strategies have been proposed to enhance the cycling stability of AFLMBs, a significant challenge remains in comparing these technologies due to inconsistent testing protocols and varying reporting metrics across studies [288]. The research direction within the scientific community regarding this field remains to be clarified. Currently, the scientific community is exploring several parallel pathways, including copper foil-based hosts, lithiophilic carbon-based hosts, and lithiophilic composite-based host. However, evaluating their readiness for commercialization requires a holistic comparison beyond just cycle life. As discussed in recent analyses, key performance metrics such as cost-effectiveness, charging speed (rate capability), and energy density must be prioritized. For instance, while some lithiophilic composite-based systems achieve long cycles, their cost and sustainability may hinder scalability. Conversely, simple copper-based current collector modifications offer cost advantages but often struggle with rate and cycle performance. Future research needs to establish standardized benchmarks to fairly evaluate which technologies best balance energy density with the cost and safety requirements needed for practical application. Thus, this section will discuss which technologies are approaching commercialization based on AFLMBs technology roadmaps, and compare key performance metrics such as cost, charge rate and energy density, as shown in Table 2.

**Table 2:** Comparison of various technical routes

Technical route	Cost	Charging speed	Energy density	Cycle life	References
Cu foil	Low	Slow	High	Short	[3]
Lithiophilic carbon host	Medium	Moderate	High	Long	[253]
Lithiophilic composite host	High	Fast	High	Long	[2]

The conventional copper foil-based method suffers from lithium dendrite growth, low active material utilization and poor battery performance. This limitation stems from the smooth, non-porous surface of the copper current collector, which fails to stably regulate lithium deposition, thereby facilitating the formation of dead lithium. Beyond electrolyte optimization and interface coating, improvement strategies include introducing a porous support structure to inhibit dendrite propagation, optimizing electrode surface modification to enhance interfacial stability, and minimizing the generation of dead lithium.

The core of lithiophilic carbon host-based strategy is to introduce carbon materials with high SSA (such as graphene, CNTs [288]) to reduce the local current density, act as a host for lithium deposition, and provide nucleation sites. Owing to the strong lithiophilicity and large SSA of these carbon materials, this strategy effectively inhibits lithium dendrite growth and enhances the uniformity of lithium deposition. Key implementation measures include carbon coating modification and the structural design of the carbon skeleton. However, the primary limitation is volume expansion resulting from non-uniform lithium deposition. This issue arises mainly from uneven pore size distribution within the carbon skeleton and inconsistent deposition rates, which lead to local lithium accumulation and structural deformation. To address these challenges, improvements can be achieved by optimizing the pore size and distribution of the carbon skeleton to enhance the uniformity of the lithiophilic coating, controlling the deposition current density to mitigate local over-deposition and incorporating lithiophilic active sites on the carbon surface to guide uniform lithium deposition, thereby alleviating volume expansion.

The core advantage of the lithiumphilic carbon-based support method lies in utilizing the porous structure and lithiophilic properties of carbon materials to reduce the nucleation overpotential of lithium deposition, suppress dendrite growth, and mitigate dead lithium formation, thereby enhancing the battery’s cycle stability. However, its practical application is limited by a complex preparation process and high production costs. Improvement strategies primarily include: optimizing the pore size distribution of the carbon host to augment its lithium storage capacity and lithiophilicity, streamlining the preparation process to lower costs, and synergizing with a compatible electrolyte system to stabilize the SEI film. These measures aim to minimize capacity fading caused by non-uniform deposition, further improving both the cycling performance and safety of the battery.

Based on the comparative analysis, we conclude that the copper foil-based approach is the most viable candidate for near-term commercialization, owing to its cost-effectiveness and mature processing technology. In the medium term, 3D lithiophilic carbon-based hosts are projected to achieve commercial breakthroughs through structural optimization, effectively balancing critical metrics such as cost and energy density. Looking further ahead, 3D lithiophilic composite hosts hold the greatest long-term potential; however, realizing this potential requires further mitigation of challenges related to manufacturing costs and process complexity.

### Benchmarking Analysis

The practical application of AFLMBs hinges on the synergistic optimization of three key parameters: high-loading cathodes, low anode-to-cathode capacity ratios (A/C ratio, also known as N/P ratio), and lean electrolyte conditions. High-loading cathodes supply sufficient cyclable lithium for low A/C ratio systems, while lean electrolyte conditions maximize the energy density gains from the former two factors. Studies show that increasing the cathode areal capacity from 2.8 to 5.0 mAh cm⁻^2^ boosts the energy density of AFLMBs from 343.0 to 452.4 Wh kg⁻^1^, with the cathode contributing 59.5% of the total energy density at an E/C ratio of 1.2 g Ah⁻^1^. Furthermore, reducing the electrolyte-to-capacity ratio from an excess of 4.0 g Ah⁻^1^ to a lean 1.2 g Ah⁻^1^ enhances energy density by approximately 33.3% [53]. However, another study using 5 Ah pouch cells reveals that increasing the electrolyte injection volume from 1.8 to 2.0 g Ah⁻^1^ extends the cycle life from 300 to 400 cycles [289]. This quantitative analysis demonstrates that while lean electrolyte conditions enhance energy density, they accelerate electrolyte depletion and interfacial degradation. Conversely, a moderate increase in electrolyte injection, despite slightly compromising energy density, can significantly extend cycle life. Therefore, the synergistic optimization of these three parameters must strike a quantitative balance between energy density and cycling stability. Through strategies such as current collector coating modification, process innovations (e.g., dry electrode technology), and electrolyte formulation optimization, the performance synergy among these factors can be achieved, fully unlocking the technological potential of AFLMBs. This synergistic system is particularly well suited for short-cycle, high-energy-output applications. Moving forward, advanced techniques such as AI-assisted electrolyte screening and composite current collector design will be essential to further refine the interplay among these parameters, enabling the transition of AFLMBs from short-cycle scenarios to long-life, large-scale applications.

## Conclusions and Future Perspectives

### Conclusions

AFLMBs are widely regarded as one of the most promising next-generation rechargeable battery technologies. By eliminating excess metallic lithium, AFLMBs not only significantly mitigate safety concerns, but also maximize gravimetric and volumetric energy density. However, the intrinsic lithiophobic nature of conventional carbon-based hosts often leads to inhomogeneous Li nucleation and dendrite growth. This challenge can be well addressed through rational 3D lithiophilic design. In this review, we first summarize various carbon-based materials suitable for AFLMBs, including graphene, CNTs, PCs, and CFs. The nucleation mechanisms and lithiophilic site modulation strategies are then elucidated. This is followed by a systematic discussion on surface modification, structural engineering, framework design, gradient design, and synergistic design of 3D lithiophilic carbon-based hosts, along with operando characterization techniques. Finally, a comprehensive practical discussion is provided regarding challenges related to scalability for mass production, cost-effectiveness, and electrolyte compatibility. We highlight that 3D lithiophilic carbon-based hosts, characterized by ample void space and superior lithiophilicity, effectively facilitate uniform Li deposition and mitigate volume expansion, leading to an significantly enhanced electrochemical performance, as summarized in Table 3, demonstrating how 3D lithiophilic carbon-based host designs can meet the application requirements of high-performance AFLMBs.

**Table 3:** Summary of overall performance for various hosts in AFLMBs

Host	CE	Energy density	Cycle life	Areal capacity (Cathode)	N/P ratio	E/C ratio	References
Li_2_ZnCu_3_	99.2% average CE (ACE) after 200 cycles @ 6mAh cm^−2^	407.4 Wh kg^−1^ (coin cells)	77.2%@130 cycles, 0.2C	4 mAh cm^−2^	3	12.5 μL mAh^−1^	[223]
CuZnMOF-Li	> 90% after 150 cycles @ 1.0 mAh cm^−2^	N/A	90%@600 cycles, 1.0C	N/A	N/A	N/A	[224]
Sn@B/N/F-CMFs	95.39% ACE after 100 cycles @ 2.0 mAh cm^−2^	494 Wh kg^−1^ (pouch cells)	90@160 cycles, 0.5C	1.10 mAh cm^−2^	3.5	2.5 g Ah^−1^	[204]
S/N-rGO/MWCNTs	94,9% ACE after 500 cycles @ 1.0 mAh cm^−2^	N/A	98.8%@150 cycles, 1.0C	N/A	N/A	N/A	[205]
Ag@CNF	98% ACE after 400 cycles @ 1.0 mAh cm^−2^	424 Wh kg^−1^ (pouch cells)	90%@300 cycles, 1.0C	1.5 mAh cm^−2^	2	3 g Ah^−1^	[211]
Au/CCC foam	> 80% ACE after 200 cycles @ 3.0 mA h cm^−2^	N/A	60%@20 cycles, 0.1C	3.8 mAh cm^−2^	0	34.2 μL mAh^−1^	[107]
LZ-rGO	98.74% ACE	N/A	97.9%@900 cycles, 3.0C	2.4 mAh cm^−2^	1.2	22.1 μL mAh^−1^	[212]
Zn/CF@NH_2_-UiO-66	98.9% ACE after 500 cycles @ 1.0 mAh cm^−2^	N/A	93.4%@1700 cycles, 2.0C	0.48–0.80 mAh cm^−2^	1.74	N/A	[213]
CP/Z6L1	98.0% ACE	N/A	85.6%@75 cycles, 2.0C	1.94 mAh cm^−2^	N/A	45 μL mAh^−1^	[214]
NCHZS@CC	98.6% ACE after 160 cycles @ 4.0 mAh cm^−2^	N/A	86.3%@900 cycles, 2.0C	1.84 mAh cm^−2^	2.17	48.6 μL mAh^−1^	[215]
LAG/LA103Z	98.6% ACE after 400 cycles @ 1.0 mA h cm^−2^	N/A	60%@120 cycles, 0.5C	3.86 mAh cm^−2^	N/A	N/A	[216]
KNCC	99.8% ACE after 400 cycles @ 2.0 mA h cm^−2^	N/A	86.0%@ 500 cycles, 1.0C	2.24 mAh cm^−2^	0	N/A	[225]
g-C_3_N_4_@Ag	98% ACE after 500 cycles @ 1.0 mA h cm^−2^	N/A	67%@ 200 cycles, 1.0C	N/A	N/A	N/A	[226]
NC/Mg@RGO	98.65% ACE after 150 cycles @ 1.0 mA h cm^−2^	N/A	86.86%@800 cycles, 1.0C	0.8 mAh cm^−2^	N/A	N/A	[120]
CG host	96% ACE after 120 cycles	359 Wh L^−1^ (coin cells)	90%@100 cycles, 1.0C	1.1 mAh cm^−2^	3.6	143 μL mAh^−1^	[231]
SiC/CC	95% ACE after 100 cycles @ 1.0 mA h cm^–2^	N/A	80%@120 cycles, 0.5C	0.72 mAh cm^−2^	4.4	295 μL mAh^−1^	[141]
GDD-CH	99.3% ACE after 500 cycles @ 0.5 mAh cm^−2^	378 Wh kg^−1^ (coin cells)	86.3%@300 cycles, 1.0C	2.16 mAh cm^−2^	1.51	N/A	[232]
GFC@PVDF	98.5% ACE after 300 cycles @ 1.0 mAh cm^−2^	N/A	83.3%@170 cycles, 1.0C	1.12–1.28 mAh cm^−2^	1.5	N/A	[235]
HEMP@RGO-MTL@PH	98.7% ACE after 200 cycles @ 3.0 mAh cm⁻^2^	414.7 Wh kg^−1^, 775.6 Wh L^−1^ (pouch cell)	90.9%@100 cycles, 0.5C	3.3 mAh cm^−2^	0.21	N/A	[140]
GZnO/CC	N/A	406.7 Wh kg^−1^ (coin cell)	90.7%@1000 cycles, 1.0C	2.22 mAh cm^−2^	1.8	N/A	[138]
GPCS	98% ACE after 320 cycles @1.0 mAh cm^−2^	N/A	93.6%@600 cycles, 0.7C	0.6 mAh cm^−2^	7.5	54.1 μL mAh^−1^	[238]
GA	N/A	N/A	35.7%@200 cycles, 2.0C	0.40 mAh cm^−2^	50	62.5 μL mAh^−1^	[239]
3D-CPA	98.8% ACE after 250 cycles	N/A	87.2%@100 cycles, 0.5C	4 mA h cm^–2^	2	N/A	[240]
GPCF	99% ACE after 160 cycles @ 1.0 mAh cm^–2^	N/A	70%@370 cycles, 0.5C	N/A	N/A	N/A	[237]
ZnO@HPC	97.6% ACE after 200 cycles @1.0 mAh cm^−2^	N/A	N/A	N/A	N/A	N/A	[242]
CF@HGL	98.8% ACE after 500 cycles @1.0 mAh cm^−2^	559.9 Wh kg^−1^	72.0%@100 cycles, 0.5C	3.86 mAh cm^−2^	0.005	4.13 g Ah^−1^	[243]
3D-AGBN	97.3% ACE after 50 cycles @ 6.0 mAh cm^−2^	N/A	31.25%@1000 cycles, 10.0C	0.28–0.30 mAh cm^−2^	40- 42.86	N/A	[244]
DLCP	98.6% ACE after 10 cycles @ 0.5 mAh cm^−2^	N/A	89.7%@400 cycles, 1.0C	1.58 mAh cm^−2^	1.26	8.4 μL mAh^−1^	[236]

### Future Perspectives

Despite significant progress in the development of AFLMBs, the use of 3D carbon-based lithiophilic hosts as current collectors still faces critical challenges. Thus, continuous design innovation and in-depth research are required to resolve these challenges.

1. The practical advancement of AFLMBs is transitioning from phenomenological description to quantitative regulation. The core challenge at present lies in transforming the inherently complex process of lithium deposition into a predictable behavior. Accordingly, we propose three scientific questions to be addressed along with their corresponding technical objectives: a) How can the relationship between the density of lithiophilic sites in carbon-based hosts and the nucleation overpotential be quantitatively established? Although the lithiophilicity of single atoms such as N and Ag has been demonstrated to effectively reduce the lithium nucleation overpotential, a precise predictive model with an error margin of less than 5% remains lacking to determine how the density and distribution of these sites govern the nucleation barrier from a quantitative perspective. Future research should integrate first-principle calculations with controlled doping experiments to evolve the concept of “lithiophilicity” from a qualitative descriptor into a quantifiable design parameter. b) How does the Li⁺ transport pathway within 3D carbon hosts influence the uniformity of lithium deposition? Insights derived from planar substrates are insufficient to meet the demands of understanding 3D carbon hosts with complex pore architectures. Therefore, monitoring the transport pathways of lithium ions within 3D frameworks using operando NMR or in situ optical microscopy is essential for elucidating the evolution mechanism of deposition uniformity and clarifying the origin of “dead lithium.” c) What are the critical conditions for the formation of lithium carbide, and how can it be avoided? Under the stringent conditions of AFLMBs, where the lithium inventory is extremely limited, irreversible loss of active lithium due to interfacial side reactions constitutes the primary cause of battery failure. Systematically determining the critical relationships among temperature, current density, and lithium carbide formation, thereby delineating its phase transition boundaries, provides a scientific foundation for defining safe and efficient charge–discharge operating windows.
2. Beyond the three specific scientific questions outlined above, future research must be anchored to three measurable, interrelated, and highly challenging technical objectives: a) CE must exceed 99.5%, which necessitates that the irreversible loss of active lithium per cycle be controlled to within 0.5%. This imposes atomic-level requirements for suppressing interfacial side reactions and eliminating “dead lithium” formation. b) Cycle life must surpass 500 cycles at a 1C rate, demanding that the electrode structure maintain high reversibility and mechanical stability over extended charge–discharge cycling, rather than exhibiting only initial-cycle excellence. c) Energy density must reach above 400 Wh kg⁻^1^, compelling researchers to strike an ultimate balance between the finite lithium inventory and the mass of host materials. Any increase in inactive components will impede the achievement of this target.
3. For AFLMBs, future transformative technologies will no longer be confined to single-dimensional optimization but are expected to achieve leapfrog development through the integration of intrinsic material intelligence and multifunctionality. The following three potential transformative technologies are proposed: a) Self-healing carbon hosts: By introducing dynamic bonding or other interactive forces into the carbon-based framework, the host can autonomously repair structural cracks or defects induced by volume expansion during cycling, thereby enhancing long-term structural integrity. b) Stimuli-responsive hosts: Leveraging electrode materials with temperature-sensitive or electrochemical sensitivity, the lithiophilicity of the carbon host can dynamically modulate in response to real-time temperature or current rate, achieving an optimal balance between dendrite suppression and reduction in the lithium nucleation overpotential. c) Multifunctional integrated hosts: An ideal 3D carbon-based host should simultaneously integrate current collection, lithium storage, and thermal management. Through such a monolithic design, AFLMBs can surpass an energy density of 400 Wh kg⁻^1^ while ensuring thermal safety and mechanical stability.
